# Resting-State Static and Dynamic Functional Abnormalities in Active Professional Fighters With Repetitive Head Trauma and With Neuropsychological Impairments

**DOI:** 10.3389/fneur.2020.602586

**Published:** 2020-12-10

**Authors:** Xiaowei Zhuang, Virendra Mishra, Rajesh Nandy, Zhengshi Yang, Karthik Sreenivasan, Lauren Bennett, Charles Bernick, Dietmar Cordes

**Affiliations:** ^1^Cleveland Clinic Lou Ruvo Center for Brain Health, Las Vegas, NV, United States; ^2^Department of Brain Health, University of Nevada, Las Vegas, NV, United States; ^3^School of Public Health, University of North Texas, Fort Worth, TX, United States; ^4^Pickup Family Neuroscience Institute, Hoag Memorial Hospital Presbyterian, Newport Beach, CA, United States; ^5^UW Medicine, Seattle, WA, United States; ^6^Department of Psychology and Neuroscience, University of Colorado, Boulder, CO, United States

**Keywords:** fighters, processing speed, psychomotor speed, repetitive head trauma, static & dynamic functional connectivity

## Abstract

Previous neuroimaging studies have identified structural brain abnormalities in active professional fighters with repetitive head trauma and correlated these changes with fighters' neuropsychological impairments. However, *functional* brain changes in these fighters derived using neuroimaging techniques remain unclear. In this study, both static and dynamic functional connectivity alterations were investigated (1) between healthy normal control subjects (NC) and fighters and (2) between non-impaired and impaired fighters. Resting-state fMRI data were collected on 35 NC and 133 active professional fighters, including 68 impaired fighters and 65 non-impaired fighters, from the Professional Fighters Brain Health Study at our center. Impaired fighters performed worse on processing speed (PSS) tasks with visual-attention and working-memory demands. The static functional connectivity (sFC) matrix was estimated for every pair of regions of interest (ROI) using a subject-specific parcellation. The dynamic functional connectivity (dFC) was estimated using a sliding-window method, where the variability of each ROI pair across all windows represented the temporal dynamics. A linear regression model was fitted for all 168 subjects, and different t-contrast vectors were used for between-group comparisons. An association analysis was further conducted to evaluate FC changes associated with PSS task performances without creating artificial impairment group-divisions in fighters. Following corrections for multiple comparisons using network-based statistics, our study identified significantly reduced long-range frontal-temporal, frontal-occipital, temporal-occipital, and parietal-occipital sFC strengths in fighters than in NCs, corroborating with previously observed structural damages in corresponding white matter tracts in subjects experiencing repetitive head trauma. In impaired fighters, significantly decreased sFC strengths were found among key regions involved in visual-attention, executive and cognitive process, as compared to non-impaired fighters. Association analysis further reveals similar sFC deficits to worse PSS task performances in all 133 fighters. With our choice of dFC indices, we were not able to observe any significant dFC changes beyond a trend-level increased temporal variability among similar regions with weaker sFC strengths in impaired fighters. Collectively, our functional brain findings supplement previously reported structural brain abnormalities in fighters and are important to comprehensively understand brain changes in fighters with repetitive head trauma.

## Introduction

Repetitive exposure to head trauma is a risk factor for various neurological disorders, such as dementia puligistica and post-traumatic parkinsonism (1–5). Neuroimaging techniques can identify both structural and functional changes in the brain and therefore have been widely used to investigate brain abnormalities related to repetitive head trauma in multiple populations [see reviews in (6, 7)]. In particular, a reduction in brain structural volumes and cortical thickness has been reported in football and ice hockey players (8, 9); abnormal white-matter organization and topological changes have been reported in soccer, football, and ice hockey players (10, 11), and functional alternations in brain intrinsic networks have been reported in football players, veterans, and mild traumatic brain injury (TBI) patients (12–16).

These studies have advanced our understanding of both structural and functional brain changes in multiple populations exposed to repetitive head trauma. Active professional fighters, who experience head hits in both games and training, are also at a high risk of potential brain damages. The Professional Fighters Brain Health Study (PFBHS) launched at our center in 2011 (17) has focused on investigating brain changes related to repetitive head trauma and their associations with clinical and neuropsychological deficits in active professional fighters. Specifically, in PFBHS, structural MRI studies have revealed both cross-sectional and longitudinal brain volumes loss in fighters (18); and in a subset of fighters with neuropsychological impairments, abnormal white matter microstructural organization (19) and white matter topological reorganizations (20) have been observed with diffusion-weighted MRI studies.

Collectively, these neuroimaging findings suggest that structural brain changes in active professional fighters may reflect clinically relevant phenomena. However, brain *functional* connectivity changes related to repetitive head trauma from neuroimaging data remain unclear in this fighters' cohort. Previous studies have demonstrated that the blood-oxygen-level-dependent (BOLD) signal measured by resting-state functional MRI (fMRI) can detect subtle changes in brain function in neurological disorders, including TBI (14, 21–24). Thus, resting-state fMRI may be a powerful tool to investigate functional changes between fighters and cognitively normal control subjects (NC), which might reveal a vulnerability related to repetitive head trauma in fighters. Furthermore, a subset of fighters in PFBHS demonstrate impaired-range processing speed (PSS) performances during visual-perception and fine-motor tasks with visual-attention and working-memory demands. Structural brain changes in impaired fighters include both volumetric loss and structural connectivity reorganizations, which have been further linked to repetitive head hits (19, 20). Investigating functional brain differences between non-impaired and impaired fighters would further advance our understanding of the flexibility and variability of functional brain changes within fighters and the neural representations of the reported structural brain abnormalities.

In resting-state fMRI analysis, functional brain changes have been widely studied using the functional connectivity (FC) matrix, in which each value represents a functional connectivity strength that is quantified by the Pearson's correlation coefficient between one brain region pair (25), and all brain region pairs are arranged accordingly [see (26) for a review]. This FC matrix, also known as the functional connectome, allows ROI-based whole-brain functional connectivity analysis without any prior selected seed regions, and both within and between network connections can be investigated. Furthermore, conventionally, the functional connectivity strength in the FC matrix is assumed to be static (sFC) and is computed using the entire fMRI time series. More recently, various studies have reported that the FC is changing periodically during the epoch of an fMRI scan, also termed as the dynamic FC [dFC, (27) (review); (28, 29) (review); (30) (review)]. Several dFC measures have been proposed such as dFC-variation (28), dFC-stability (31) and dFC-states (32). These measures have been demonstrated to reflect temporal dynamics of neural activities detected in electrophysiological recordings (33, 34) and to reveal alterations in normal development and various neurological disorders (14, 35–37). Therefore, in addition to sFC, it is also critical to evaluate dFC in this well-characterized active professional fighters' cohort, which might uncover if a temporal dependence exists in FC changes related to repetitive head trauma.

In summary, in this study, using resting-state fMRI data, we first explored whether there were any differences in sFC or dFC matrices between NC and all active professional fighters to investigate brain functional changes related to repetitive head trauma. We also compared both sFC and dFC matrices between non-impaired and impaired fighters to determine brain functional changes related to impaired performances on PSS tasks with visual-attention and working-memory demands. We further performed an association analysis using all fighters to evaluate functional brain changes related to PSS task performances without creating fighters' group divisions. Results from our functional analyses will add supplementary knowledge of functional brain changes in this fighters' cohort to previously reported structural brain deficits.

## Methods

Both active professional fighters and NC subjects were recruited from the PFBHS at our center (17). The PFBHS was approved by the Cleveland Clinic Institutional Review Board, and written informed consent was obtained from all participants. The protocols of the experiment were explained to all subjects and were performed according to the Declaration of Helsinki guidelines and Belmont Report.

252 healthy professional fighters and 35 NC subjects were recruited from 2011 to 2016 at our center. Fighters with either a current or prior psychiatric or neurological disorder, are younger than 18 years old, or who have participated in a sanctioned competition within 45 days before their MRI visits were excluded. Detailed demographics including age, sex, years of education (YOE), and race; fighting histories, including the number of fights (NOF), years of fighting (YOF) and knock-out (KO) histories were recorded for 221 fighters. NC subjects were healthy and without any history of contact sports at high school or above levels.

### Binary Division of Fighters Into Impaired and Non-Impaired Groups

For each fighter, CNS Vital Signs tests (38), including the finger-tapping task, symbol digit coding task, Stroop task, and verbal memory task, were performed on the same day their MRI data were collected and were used to evaluate their processing-speed, visual-attention, and verbal memory cognitive functioning. PSS score is calculated as number of correct responses on the symbol digit coding task minus number of incorrect responses, and psychomotor speed (PSY) score is calculated using bilateral finger tapping performances and number of correct responses on the symbol digit coding task. Both PSS and PSY reflect an individual's performance on visual-perception and fine-motor tasks with visual-attention and working-memory demands (CNS Vital Signs Guide, www.CNSVS.com). Based on these two scores, fighters were divided into impaired and non-impaired groups.

Previous studies have reported structural brain damage specifically in impaired fighters (19, 20, 39). We were also interested in functional brain changes in this same cohort. Therefore, the same criteria were applied to divide fighters into impaired and non-impaired groups as in Mishra et al. (20). Briefly, raw scores of PSS and PSY were first standardized by converting to z-scores, and those fighters who had both or either standardized PSS and PSY two standard deviations below the average of the age and education matched general populations (not fighters) were identified as impaired fighters. Under these criteria, 70 impaired fighters and 70 statistically demographically matched non-impaired fighters, previously used in Mishra et al. (20), were included in our analysis.

### MRI Data Acquisition and Final Subjects

#### MRI Data Acquisition

MRI data were collected on a 3T Siemens Verio scanner with a 32-channel head coil. Resting-state fMRI data were collected with the following parameters: TR 2,800 ms, TE 28ms, flip angle 80 degrees, in-plane resolution 2 × 2 mm, slice thickness 4 mm, 30 axial slices, and 137 time frames. Additionally, a high resolution T1-weighted structural image was acquired using a 3D MPRAGE sequence with the following parameters: TR 2,300 ms, TE 2.98 ms, TI 900 ms, flip angle 9 degrees, in-plane resolution 1 × 1 mm, and slice thickness 1.2 mm.

Two impaired fighters and five non-impaired fighters were removed due to missing or incomplete fMRI scans, leading to 68 remaining impaired fighters, 65 non-impaired fighters and 35 NC subjects in our analysis.

#### Comparisons of Demographic Variables

Group differences for each demographic and neuropsychological variable were assessed between NC and all fighters and between non-impaired and impaired fighters. A Chi-square test was used to test the significance of categorical variables, and a two-sample *t*-test was used to determine the significance of continuous variables. Statistical significance was established at a *p*-value of 0.05 for each test.

### MRI Data Analysis

**Figure 1:**
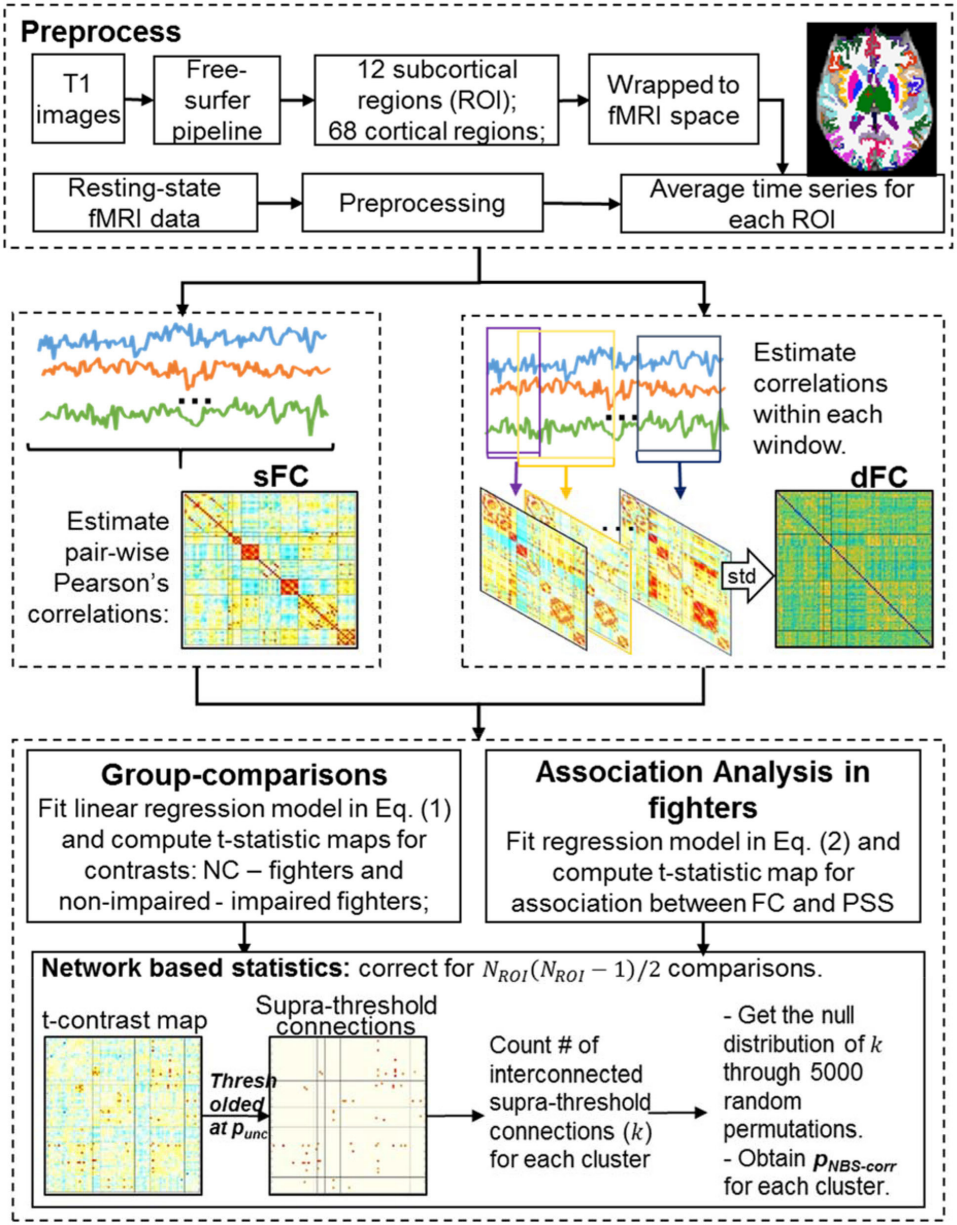
MRI data analysis.

Figure 1 illustrates the processing steps of MRI data. In the following, we explain each step in detail.

#### Structural Image Analysis

As shown in Figure 1 (top), each T1-weighted image was first segmented into gray matter (GM), white matter (WM), and cerebrospinal fluid (CSF) using the SPM12 toolbox (http://www.fil.ion.ucl.ac.uk/spm/). Binarized WM and CSF masks were then generated and co-registered to the subject fMRI space using 12 parameters affine transformation in Advanced Normalization Tools (ANTs) software (http://stnava.github.io/ANTs/). Each T1-weighted image was input to the FreeSurfer 6.0 processing pipeline (40) to generate subject-specific anatomical labeling from the Desikan-Killiany atlas (41) including subcortical labeling, yielding 80 ROIs for every subject (i.e. *N*_*ROI*_ = 80). Labels, abbreviations, and corresponding brain lobes of each ROI are listed in Supplementary Table 1.

#### Functional Image Analysis

##### Pre-processing

The first 4 time frames (~12 s) were removed to allow the MR signal to achieve T1 equilibrium. Remaining time frames were slice-timing corrected and realigned to the mean echoplanar image in SPM12 (http://www.fil.ion.ucl.ac.uk/spm/). FMRI data were further spatially smoothed using a 6 mm 3D-Gaussian filter in the subject fMRI space. To remove motion-related and physiological noises in fMRI data, additional preprocessing steps, including nuisance regression and high-pass filtering, were applied to the smoothed fMRI data. A high-pass cosine filter with a cut-off frequency of 0.008 Hz was applied first. Nuisance regressors included six head motion parameters obtained from the realignment step and WM and CSF signals generated using the CompCor method (42). Specifically, binarized WM and CSF masks in subject-fMRI space were first eroded with 2 voxels to ensure that only pure WM and CSF voxels would be included in the mask. WM and CSF fMRI signals were then extracted for each subject using eroded masks. The first 5 principal components of WM signals and the first 5 principal components of CSF signals were used as nuisance regressors from the CompCor method. Finally, all voxel time courses were variance normalized.

##### FMRI motion

A root-mean-square (RMS) head motion for each subject following Power et al. (43) was computed. Specifically, rotational displacements first were converted to translational displacements by projection to a surface of a 50 mm radius sphere. RMS head motion was then computed from both the original translational displacements and the converted rotational displacements. Between-group differences for RMS head motion were assessed using two-sample *t*-tests for both NCs vs. all fighters and non-impaired vs. impaired fighters.

##### sFC matrix

A sFC matrix was constructed for each subject by computing pair-wise Pearson's correlation values among all 80 ROIs [Figure 1 (middle left)]. Specifically, subject-specific ROI labeling in T1 space obtained in the structural image analysis section was transformed to subject fMRI space using the 12 parameters affine transform in ANTs software (http://stnava.github.io/ANTs/). Average time signals were obtained for each ROI, and the Pearson's correlation was computed for every ROI pair using the entire time series, leading to an 80 × 80 matrix that represents the sFC. Finally, correlation values were converted to Fisher's z scores (44) to ensure a Gaussian-like distribution.

##### dFC matrix

A dFC matrix was constructed for every subject using a sliding-window approach (32), where Pearson's correlations of every ROI-pair were computed within each window (Figure 1(middle right). A single-scale time-dependent (SSTD) window-size was computed for every ROI-pair at every time point for every subject (37, 45), and all windows were slid over 1 TR (2.8 s). For every ROI-pair, the standard deviation of correlation values across all windows was then used as a summary index of the temporal variability. A larger value of this index indicated less stable and more varying functional connections between two ROIs. This summary index for every ROI-pair formed the dFC matrix.

### Statistical Analysis

#### Group Comparisons

For each value in the sFC or dFC matrix, one general linear regression model was used for all subjects:

(1) $$\begin{array}{l} {\text{y}}_{ij}={\text{x}}_{NC}\beta_{\text{NC}}+{\text{x}}_{\text{non}}\beta_{\text{non}}+{{\text{x}}_{\text{im}}\beta }_{\text{im}}+{\text{X}}_{\text{covariates}}\beta_{\text{covariates}}+\varepsilon \end{array}$$

where ${\;\text{y}}_{ij}\in {\mathbb{R}}^{168\times 1}$ is a vector with sFC or dFC values between region *i* and region *j* from all subjects; ${\text{x}}_{NC}\in {\mathbb{R}}^{168\times 1},\;{\text{x}}_{non}\in {\mathbb{R}}^{168\times 1}$ and ${\text{x}}_{im}\in {\mathbb{R}}^{168\times 1}$ are dummy coding vectors of NCs, non-impaired fighters and impaired fighters, respectively, with 1 representing group assignment and 0 otherwise. The covariates matrix ${\text{X}}_{covariates}\in {\mathbb{R}}^{168\times 5}$ includes vectors of age, sex, YOE, races, and resting-state fMRI RMS-motions, and **ε** ∈ ℝ^168×1^ is the residual vector.

T-statistics were computed for the contrast where NC have larger values than fighters using the contrast vector: ${\text{c}}_{1}={\left[1,-0.5,-0.5,\;0\right]}^{T}\in {\mathbb{R}}^{8\times 1}$ and for the contrast where non-impaired fighters have larger values than impaired fighters using the contrast vector ${\text{c}}_{2}={\left[0,1,-1,\;0\right]}^{T}\in {\mathbb{R}}^{8\times 1}$. Signs of every contrast map were also reversed for contrasts where NC have smaller values than fighters, and for non-impaired fighters have smaller values than impaired fighters.

The effect size in terms of Cohen's *d* for each contrast was also estimated as: *d* $=\frac{{\text{c}}_{i}^{T}\hat{\beta }}{std_{pool}},\;i=1,2$, where $\hat{\beta }\in {\mathbb{R}}^{8\times 1}\;=$ ${\left(\text{X}\right)}^{+}{\text{y}}_{ij}$ was the estimated β coefficient from Equation (1). (**X**)^+^ was the pseudo-inverse of **X**, and *std*_*pool*_ was the pooled standard deviation of all 168 subjects after adjustment for the covariates. Since **x**_*NC*_, **x**_*non*_ and **x**_*im*_ were vectors with 1 representing each group assignment and 0 otherwise, *std*_*pool*_ can be further estimated as $std_{pool}=\sqrt{\frac{{\left({\text{y}}_{ij}-\text{X}\hat{\beta }\right)}^{2}}{df\varepsilon }}$, where *dfε* was the degree of freedom of the residual term in Equation (1).

#### Associations Between FC Measures and PSS Scores in All Fighters

To further determine sFC and dFC changes related to impaired performances on PSS tasks with visual-attention and working-memory demands in fighters without creating any *artificial* group-division, a general linear model analysis was implemented between each sFC or dFC measure (**y**_*ij*_) and PSS scores across all 133 fighters, with age, sex, YOE, races and resting-state fMRI RMS-motion as covariates:

(2) $$\begin{array}{l} {\text{y}}_{ij}=\beta_{0}+\beta_{1}\times \text{PSS}+\beta_{2}\times \text{Age}+\beta_{3}\\ \;\times \text{Sex}+\beta_{4}\times \text{YOE}+\beta_{5}\times \text{Race}+\beta_{6}\times \text{Motion} \end{array}$$

T-statistics for the slope of each sFC or dFC measure against PSS (β_1_) was computed and used to evaluate the associations between FC measures and PSS scores.

#### Network-Based Statistics Correcting for Multiple Comparisons in the Graph

For the sFC or dFC matrices, a total of $\frac{N_{ROI}\left(N_{ROI}-1\right)}{2}=3,160$ unique comparisons were conducted for the whole connectome. To evaluate significant between-group differences of FC measures and significant associations between FC measures and PSS scores from this whole connectome, we used the network-based statistics (NBS) method to adjust for the multiple comparisons (46). NBS non-parametrically determines the cluster-wise corrected statistics through comparing the true statistical map with the null statistical maps generated by random-permutations (46). Therefore, NBS non-parametrically controls the family-wise error rate in the weak sense, when mass-univariate testing is performed at every connection comprising a graph.

More specifically, an initial uncorrected *p*-value (p_unc_) threshold was first applied to the true t-statistics map to identify a set of supra-threshold connections [Figure 1(bottom)]. Interconnected supra-threshold connections were then identified as clusters, and numbers of connections (*k*) within each cluster were stored. Next, 5,000 random permutations were performed on group-assignments of sFC or dFC matrices, and the same models in Equation (1, 2) were fitted for each permutation. T-statistics maps were computed for the same contrasts (or the association) for each permutation. The same p_unc_ threshold used in thresholding the true contrast map was applied to determine the interconnected supra-threshold clusters in the null contrast maps. The maximal cluster size of each permutation formed the empirical null distribution of the maximal cluster size. The NBS-corrected *p*-values (p_NBS−corr_) of stored cluster size *k* was finally obtained by finding the percentile of *k* in the cumulative null distribution of the maximal cluster size distribution. Multiple p_unc_ were used in our analysis to determine significant NBS clusters with different initial p-thresholds.

## Results

### Demographics and fMRI Motion Comparisons

Detailed demographics for 35 NC and 133 fighters, consisting of 68 impaired fighters and 65 non-impaired fighters, are outlined in Table 1. Values are reported as the mean ± standard deviation for each variable. Statistical significance (*p*-values) for comparisons of NC vs. fighters and non-impaired vs. impaired fighters are reported in Tables 1A,B, respectively.

**Table 1:** Subject demographics.

**A. COMPARISONS BETWEEN NC AND ALL FIGHTERS**			
	**Control Subjects**	**Fighters**	**NC vs. Fighters**
No. of subjects	35	133	–
Sex	31 Men, 4 Women	123 Men, 10 Women	0.46
Age at imaging	28.80 ± 8.52	29.29 ± 5.77	0.69
Years of education	14.37 ± 2.57	13.15 ± 1.89	**0.002**
Race			
Unknown	2	19	**<0.001**
Pacific islander	2	7	
American Indian/Alaskan Native	0	3	
Asian	5	1	
African American	3	46	
White	23	57	
Processing speed score	63.63 ± 18.18	49.37 ± 11.73	**<0.001**
Psychomotor speed score	193.94 ± 25.61	167.80 ± 21.94	**<0.001**
Number of fights	–	14.46 ± 12.77	–
Years of fighting (years)	–	5.45 ± 4.25	–
Knock-outs	–	0.93 ± 1.53	–
fMRI motion (mm)	0.27 ± 0.09	0.24 ± 0.10	0.12
**B. COMPARISONS BETWEEN NON-IMPAIRED AND IMPAIRED FIGHTERS**			
	**Non-impaired fighters**	**Impaired fighters**	**Non-impaired vs. impaired fighters**
No. of Subjects	65	68	-
Sex	58 Men, 7 Women	65 Men, 3 Women	0.16
Age at imaging	28.78 ± 5.27	29.78 ± 6.20	0.32
Years of education	13.28 ±1.63	13.03 ± 2.12	0.45
Race			
Unknown	8	11	0.46
Pacific islander	5	2	
American Indian/Alaskan Native	1	2	
Asian	0	1	
African American	26	20	
White	25	32	
Processing speed score	58.28 ± 7.30	40.85 ± 8.34	**<0.001**
Psychomotor speed score	183.12 ± 15.95	153.16 ± 16.11	**<0.001**
Number of fights	14.45 ± 12.97	14.47 ± 12.68	0.99
Years of fighting (years)	5.03 ± 4.02	5.85 ± 4.45	0.27
Knock-outs	0.78 ± 1.14	1.07 ± 1.83	0.27
fMRI motion (mm)	0.23 ± 0.09	0.25 ± 0.11	0.3

*Statistically significant differences (p < 0.05) are highlighted in bold*.

As listed in Table 1, age and sex are matched between NC and fighters and between non-impaired and impaired fighters. YOE are significantly different between NC and fighters (*p* = 0.002) but are matched between non-impaired and impaired fighters (*p* = 0.45). Race is also significantly different between NC and fighters (*p* < 0.001) but are matched between non-impaired and impaired fighters (*p* = 0.46). No significant differences in fighting histories, including NOF, YOF, and KO histories are observed between non-impaired and impaired fighters. All subjects have <0.8 mm (0.25±0.17 mm on average, max: 0.76 mm) RMS head motion during resting-state fMRI. RMS head motions are not significantly different between NC and fighters (*p* = 0.12) or between non-impaired and impaired fighters (*p* = 0.30).

Both PSS and PSY scores from the finger tapping and symbol digit coding tasks in CNS Vital Signs are significantly different between NC and fighters (*p* < 0.001), and between non-impaired and impaired fighters (*p* < 0.001). Ten other scores output from the verbal memory and the stroop tasks in the CNS Vital Signs were also included in Supplementary Table 2. Significant differences between non-impaired and impaired fighters were further observed in reaction time measures.

### Functional Brain Changes Between Groups: NBS Results

Table 2 shows the NBS corrected results for between-group comparisons with different initial uncorrected p-thresholds (p_unc_ < 0.001; 0.005; 0.01 and 0.05). The NBS corrected *p*-values (p_NBS−corr_) for contrasts NC greater than fighters and non-impaired fighters greater than impaired fighters are listed. The number of sFC connections within each significant NBS cluster (p_NBS−corr_ < 0.05) and dFC connections within each NBS cluster with p_NBS−corr_ < 0.10 are also included in Table 2. No significant NBS cluster is retained for the reversed contrast (NC less than fighters, or non-impaired fighters less than impaired fighters) with either sFC or dFC measures.

**Table 2:** NBS-sFC and dFC group comparison results: NBS corrected *p*-values (p_NBS−corr_) for each NBS cluster using different uncorrected *p*-values (p_unc_) as initial thresholds.

**sFC**	**Uncorrected *p*-values (p_unc_)**			
	**0.001**	**0.005**	**0.01**	**0.05**
NC vs. Fighters	**0.04 (8)**	**0.02 (48)**	**0.01 (99)**	**0.004 (402)**
Non-impaired vs. Impaired fighters	**0.03 (10)**	**0.02 (45)**	**0.02 (86)**	**0.02 (328)**
**dFC**				
NC vs. Fighters	0.32	0.51	0.23	0.27
Non-impaired vs. Impaired fighters	0.38	0.09 (21)	0.10 (50)	0.09 (243)

*In the parenthesis, numbers of FC connections in the sFC NBS cluster with p_NBS−corr_ <0.05 (highlighted in bold) and dFC NBS cluster with p_NBS−corr_ <0.10 are listed for each contrast*.

As shown in Table 2, for both contrasts, significant sFC NBS clusters are always retained irrespective of the initial uncorrected p-threshold used. At a strict initial uncorrected p-threshold (p_unc_ < 0.001), a small number of sFCs (8 and 10) with large effect-sizes (minimum *d* = 0.63 and 0.57) remain in the significant cluster; whereas at a loose initial uncorrected p-threshold (p_unc_ < 0.05), a large number of sFCs (402 and 328) with small effect-sizes (minimum *d* = 0.26 and 0.26) are included in the significant cluster. With an initial uncorrected p-threshold of p_unc_ < 0.005, sFC within the significant NBS cluster still demonstrate at least a medium effect-size (minimum *d* = 0.53 and 0.46). Therefore, we report significant NBS results using the initial threshold p_unc_ < 0.005 in the main manuscript. We also provide NBS results with the initial threshold p_unc_ < 0.001 in the (Supplementary Figures 1, 3).

#### sFC and dFC Changes in Fighters With Repetitive Head Trauma: NC vs. Fighters

##### sFC

**Figure 2:**
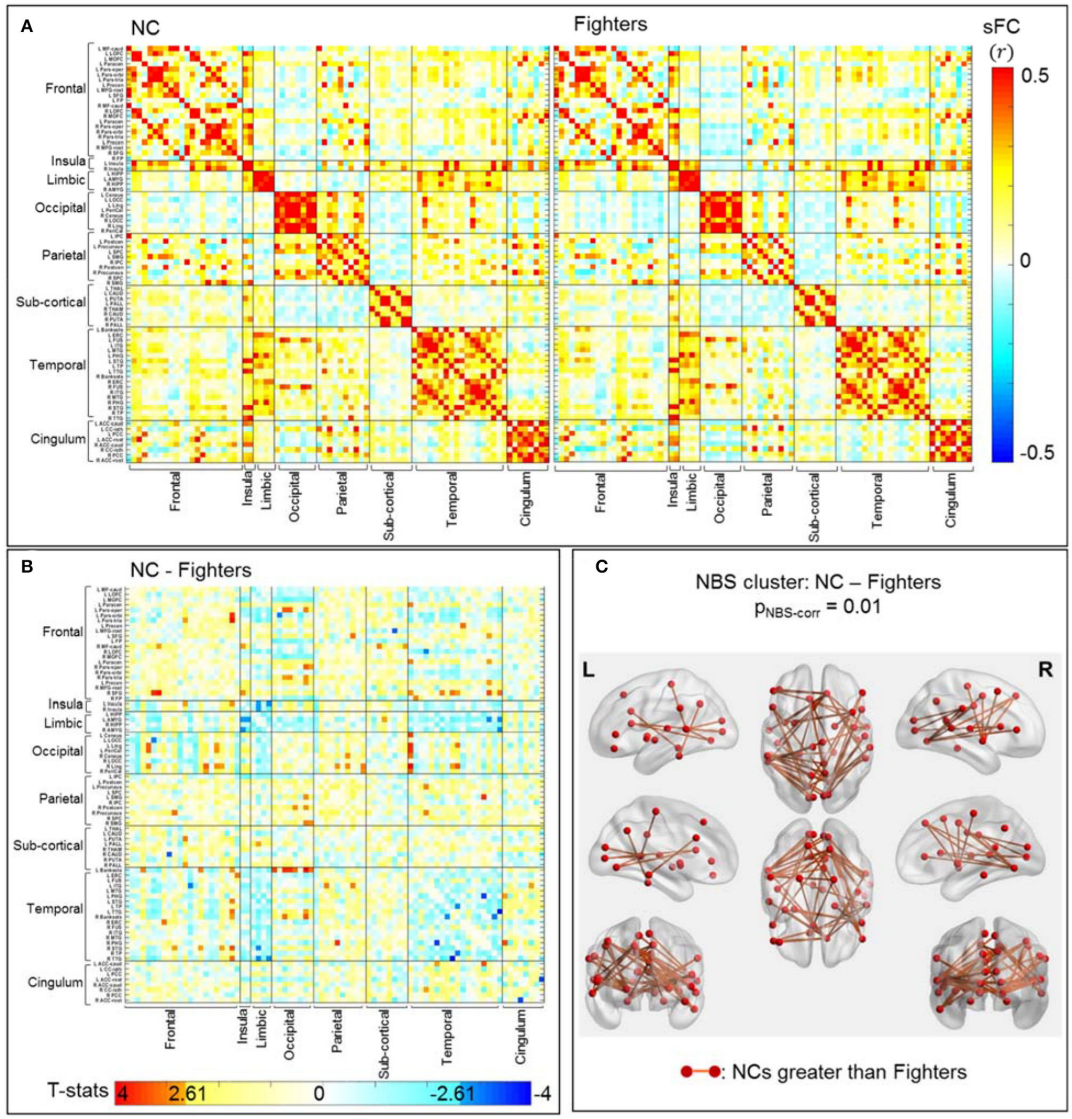
sFC comparisons between NC and fighters. **(A)** Average sFC matrices [pair-wise correlation values (*r*)] for NCs and fighters. **(B)** Uncorrected t-statistic map for contrast **c**_1_: NCs-fighters. Uncorrected *p*-value p_unc_ <0.005 is used in NBS step1 to form supra-threshold clusters for contrast NC greater than fighters, and fighters greater than NC, respectively. **(C)** Stronger connections within the significant NBS cluster in NCs than in fighters, after correction for multiple comparisons (p_NBS−corr_ = 0.01). These are mainly frontal-occipital, frontal-temporal, temporal-occipital, and parietal-occipital connections. The red circles and sticks represent the nodes and edges connecting the nodes, respectively. The nodes and edges are visualized on the Desikan–Killiany template, the same as both axis in **(A,B)**. Images are shown in neurological convention.

Average sFC matrices of NC and all fighters are shown in Figure 2A. Uncorrected t-statistic map for contrast NC vs. fighters is shown in Figure 2B. Using the initial threshold of p_unc_ < 0.005 in NBS, only one significant cluster with 48 sFC connections is observed for contrast: values for NCs are larger than for fighters, with p_NBS−corr_ = 0.01 (Figure 2C). No significant NBS cluster is retained for the reversed contrast (values for fighters larger than for NCs).

Figure 2C represents 48 sFC connections within the significant NBS cluster that are stronger in NCs than in fighters using the BrainNet Viewer (47). As listed in Table 3 (left), these paths span both cortical and subcortical regions, including mainly long-range frontal-temporal (22.92%), frontal-occipital (18.75%), occipital-temporal (18.75%) and occipital-parietal connections (10.42%). Furthermore, 33.33% connections involve default mode network (DMN) regions such as cingulate cortex, precuneus, parahippocampal regions, and middle and inferior frontal gyrus. Medium effect sizes (Cohen's *d* from 0.53 to 0.78) are observed for all connections.

**Table 3:** Significant NBS-sFC group comparisons results using uncorrected *p*-value of 0.005 (p_unc_ =0.005): list of fractions of lobe-lobe connections within the significant NBS cluster that showed stronger sFC values in NCs than in fighters (p_NBS−corr_ =0.01, *Left*) and in nonimpaired fighters than in impaired fighters (p_NBS−corr_ =0.02, *Right*).

**NC greater than fighters**		**Non-impaired fighters greater than impaired fighters**	
**Lobe-Lobe Connections**	**Fraction %**	**Lobe-Lobe Connections**	**Fraction %**
Temporal-Frontal	22.92	Temporal-Frontal	28.89
Occipital-Frontal	18.75	Limbic-Frontal	13.33
Temporal-Occipital	18.75	Parietal-Occipital	13.33
Parietal-Occipital	10.42	Temporal-Temporal	13.33
Frontal-Frontal	6.25	Temporal-Limbic	8.89
Temporal-Parietal	6.25	CingulateCortex-Parietal	6.67
CingulateCortex-Temporal	6.25	CingulateCortex-Occipital	4.44
Insula-Frontal	4.17	Temporal-Parietal	4.44
Sub-cortical-Frontal	4.17	Sub-cortical-Frontal	2.22
Parietal-Frontal	2.08	CingulateCortex-Frontal	2.22
		Parietal-Parietal	2.22

Using a stricter initial threshold of p_unc_ < 0.001 in NBS, 8 sFC connections (all included in the 48 sFC connections in Figure 2C) with a minimum effect size of *d* = 0.63 consist of the significant NBS cluster (Supplementary Figure 1). These connections are mainly frontal-occipital (37.5%) and occipital-temporal (62.5%) paths. Detailed 48 paths within the significant NBS cluster in Figure 2C, and their corresponding effect sizes and uncorrected *p*-values are listed in Supplementary Table 3, with the 8 connections within the significant NBS cluster in Supplementary Figure 1 (with p_unc_ < 0.001) highlighted in bold.

##### dFC

Average SSTD window-sizes determined by the sliding-window approach are 33.80±2.96 s for NCs and 34.42±2.29 s for all fighters, which are close to the window-size recommended in Zalesky and Breakspear (48).

After correction for multiple comparisons using NBS, we do not observe any dFC cluster with statistically significant differences between NCs and all fighters. The most significant cluster remains at p_NBS−corr_ = 0.23 (Table 2). Average dFC matrices NCs and all fighters, and the uncorrected t-statistic map for contrast NC vs. fighters are shown in the Supplementary Figure 2.

#### sFC and dFC Changes in Impaired Fighters: Non-Impaired vs. Impaired Fighters

##### sFC

**Figure 3:**
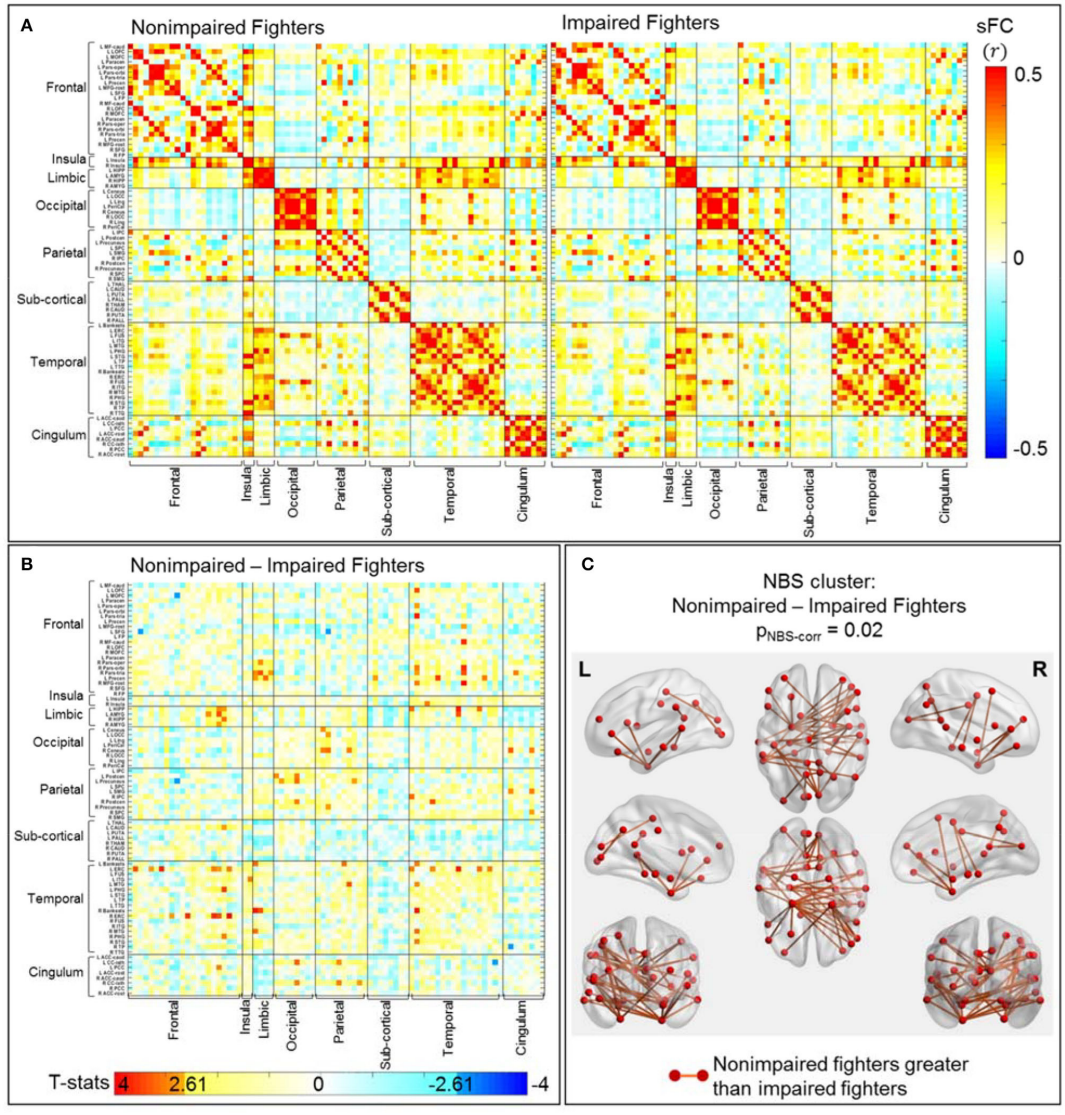
sFC comparisons between non-impaired and impaired fighters. **(A)** Average sFC matrices [pair-wise correlation values (*r*)] for nonimpaired and impaired fighters. **(B)** Uncorrected *t*-statistic map for contrast **c**_2_ : nonimpaired fighters-impaired fighters. Uncorrected *p*-value p_unc_ < 0.005 is used in NBS step1 to form supra-threshold clusters for contrast non-impaired greater than impaired fighters, and impaired fighters greater than non-impaired fighters in NBS, respectively. **(C)** Stronger connections within the significant NBS cluster (p_NBS−corr_ = 0.02) in non-impaired fighters than in impaired fighters. These are mainly temporal-frontal, limbic-frontal, and temporal-temporal connections, with regions involved in executive functions. The red circles and sticks represent the nodes and edges connecting the nodes, respectively. The nodes and edges are visualized on the Desikan–Killiany template, the same as both axis in **(A,B)**. Images are shown in neurological convention.

Separate average sFC matrices for non-impaired and impaired fighters are shown in Figure 3A. Uncorrected t-statistical map for contrast: non-impaired fighters vs. impaired fighters is shown in Figure 3B. Using the initial threshold of p_unc_ < 0.005 in NBS, one cluster with 45 sFC connections remains significantly stronger in non-impaired fighters than in impaired fighters, with p_NBS−corr_ = 0.02; whereas no cluster with stronger connections in impaired fighters than in non-impaired fighters is observed.

Figure 3C plots these 45 sFC connections within the significant NBS cluster using the BrainNet Viewer (47). As listed in Table 3 (right), these significant paths comprise mostly cortical connections, including frontal-temporal connections (28.89%), limbic-frontal connections (13.33%), parietal-occipital connections (13.33%), temporal-temporal connections (13.33%) and temporal-limbic connections (8.89%). 68.88% of these connections involve limbic or temporal regions, including the hippocampus, parahippocampal gyrus, amygdala, entorhinal cortex, and middle temporal gyrus; and 46.67% connections involve frontal regions such as the middle frontal gyrus, inferior frontal gyrus, and orbital-frontal cortex.

Using a stricter initial uncorrected p-threshold of p_unc_ < 0.001 in NBS, 10 sFC connections (all included in the 45 sFC connections in Figure 3C) with a minimum effect size of *d* = 0.57 remain in the significant NBS cluster (Supplementary Figure 3). These connections are mainly temporal-frontal (60%), temporal-limbic (20%), limbic-frontal (10%), and temporal-temporal (10%) paths. Detailed 45 connections within the significant NBS cluster in Figure 3C, their corresponding effect sizes and uncorrected *p*-values are listed in Supplementary Table 4, with the 10 connections within the significant NBS cluster in Supplementary Figure 3 (with p_unc_ < 0.001) highlighted in bold.

##### dFC

Average SSTD window-sizes determined in the sliding window approach are 34.66±2.29 s for non-impaired fighters and 34.20±2.29 s for impaired fighters. Using the initial threshold of p_unc_ < 0.005 in NBS, no significant NBS cluster (p_NBS−corr_ < 0.05) with differences between non-impaired and impaired fighters is retained. The most significant NBS cluster remains at a trend level of p_NBS−corr_ = 0.08. In this cluster, 21 paths show higher temporal variabilities in impaired fighters than in non-impaired fighters, involving regions in the temporal (e.g., entorhinal cortex, middle temporal gyrus, and parahippocampal gyrus), parietal (e.g., precuneus, paracentral gyrus), sub-cortical (pallidum and putamen), frontal (paracentral gyrus), and cingulate regions. Detailed connections within this cluster are included in the Supplementary Figure 4 and Supplementary Table 5.

### Brain Functional Changes Associated With Impaired Performances on PSS Tasks in All Fighters

Table 4A lists the NBS-corrected results of the significant positive associations between sFC and PSS scores for all fighter without the impairment group division. Results with multiple initial uncorrected p-thresholds (p_unc_ < 0.001; 0.005; 0.01 and 0.05) in NBS are listed. As shown in Table 4A, significant associations always retained after non-parametric corrections in NBS irrespective of the initial p_unc_ threshold. There is no significant NBS cluster with the negative association between sFC and PSS, or between dFC and PSS.

**Table 4A:** NBS-sFC association with PSS results with all fighters: NBS-corrected p-values (p_NBS−corr_) for each significant NBS cluster using different uncorrected p-values (p_unc_) as initial thresholds.

	**Uncorrected *p*-values**			
	**0.001**	**0.005**	**0.01**	**0.05**
Positive association between sFC and PSS	0.01 (15)	0.03 (40)	0.02 (85)	0.02 (315)

*In the parenthesis, the number of sFC edges in the significant NBS cluster is listed*.

**Figure 4:**
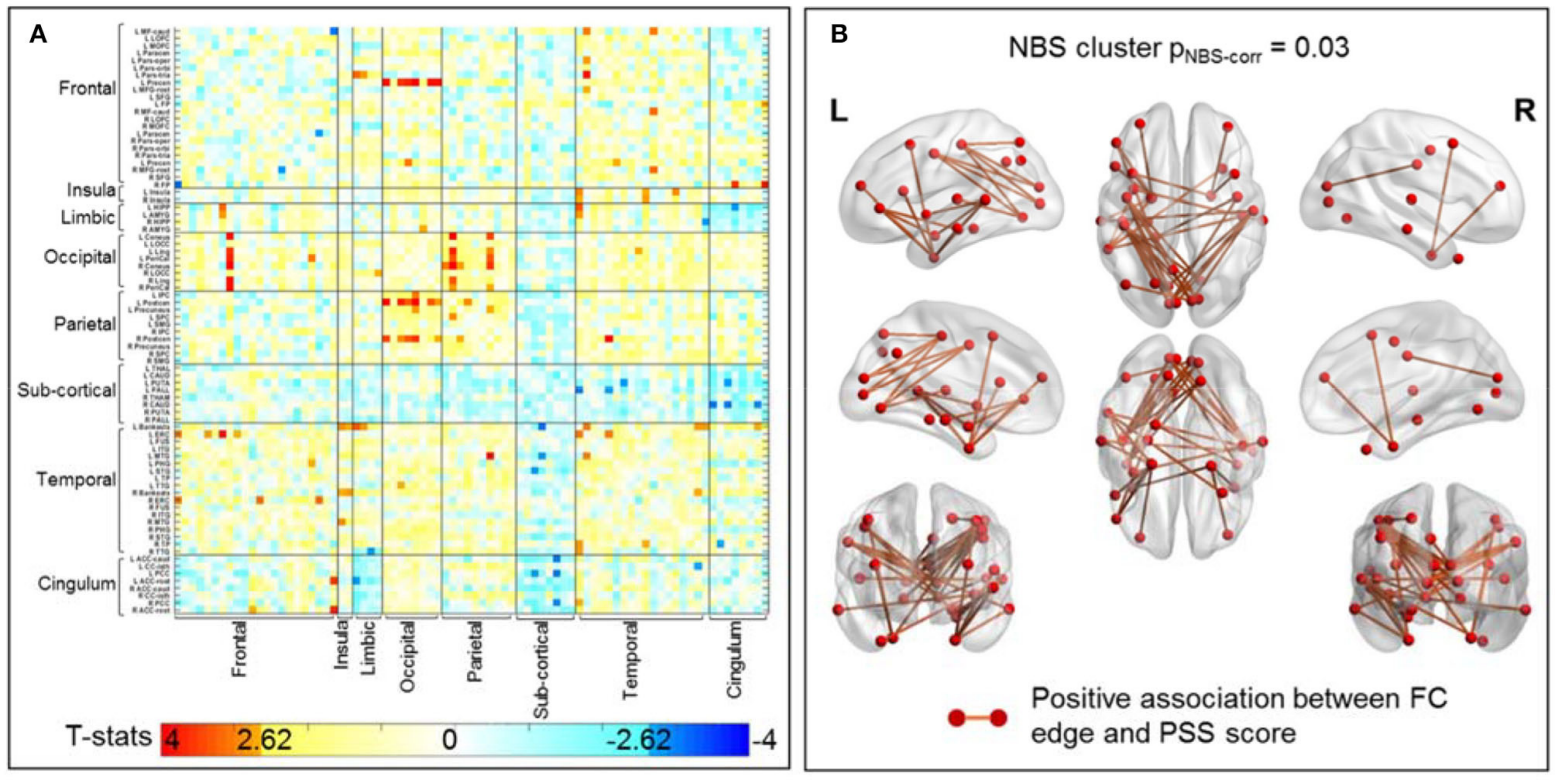
sFC association with PSS score in all 133 fighters. **(A)** Uncorrected t-statistic map for association between sFC and PSS score. Uncorrected *p* < 0.005 is used in NBS step1 to form supra-threshold clusters for the positive and negative associations separately. **(B)** sFC connections within the significant NBS cluster (p_NBS−corr_ = 0.03) that shows positive association between sFC and PSS scores. These are mainly temporal-frontal, temporal-temporal, parietal-occipital connection, with regions involved in visual-perception and executive functions. The red circles and sticks represent the nodes and edges connecting the nodes, respectively. The nodes and edges are visualized on the Desikan–Killiany template. Images are shown in neurological convention.

Figure 4A plots the uncorrected t-statistics map for this association. Using the initial threshold of p_unc_ < 0.005, one significant cluster with 40 sFC connections is retained after NBS correction (Figure 4B). As further detailed in Table 4B, these connections are mostly parietal-occipital (30%), temporal-frontal (20%), frontal-occipital (17%), and temporal-temporal (10%) connections. More importantly, 13 of these sFC connections that are significantly associated with PSS are also significantly different between non-impaired and impaired fighters (Figures 3C, 4B). These 13 sFC are frontal-temporal (5), temporal-occipital (4), temporal-temporal (3), and parietal-temporal connections. Details of these 40 sFC connections are listed in Supplementary Table 6, with 15 sFC connections within the significant NBS cluster with p_unc_ < 0.001 highlighted in bold. Common sFC connections between association analysis and group-comparison analysis are also indicated in Supplementary Table 6.

**Table 4B:** List of fractions of lobe-lobe connections within the significant NBS cluster with p_unc_ =0.005.

**Lobe-Lobe Connections**	**Fraction %**
Parietal-Occipital	30.00
Temporal-Frontal	20.00
Occipital-Frontal	17.50
Temporal-Temporal	10.00
Limbic-Frontal	5.00
Temporal-Insula	5.00
Temporal-Limbic	5.00
Temporal-Occipital	2.50
Parietal-Parietal	2.50
Temporal-Parietal	2.50

## Discussion

This study explores abnormalities in both sFC and dFC related to repetitive head trauma in a cohort of active professional fighters using resting-state fMRI data.

### Static Functional Brain Changes in Fighters

In fighters, our analysis identifies significantly weaker long-range sFC connections, as compared to NC, which are mainly related to the occipital lobe and include frontal-occipital, temporal-occipital, and parietal-occipital paths (Table 3 (left) and Figure 2C). These frontal-occipital and temporal-occipital connections are with large effect sizes and are also retained in the significant cluster when using a stricter initial uncorrected p-threshold in NBS (Table 2 and Supplementary Figure 1). Previous structural analyses in multiple populations experiencing repetitive head trauma have reported reduced white matter integrities among these distant regions, such as reduced fractional anisotropy (FA) and diffusivity of forceps major, inferior longitudinal fasciculus [one of the major occipito-temporal association tracts (49)] in contact sports athletes (10, 11, 19); and abnormal FAs of corpus callosum, superior and inferior longitudinal fasciculus, and fronto-occipital fasciculus in mild TBI patients [(50) (review)]. These white matter structural abnormalities related to repetitive head trauma might underlie our disrupted functional connections among these distant regions in fighters, who also experience multiple head hits.

In other populations experiencing repetitive head trauma, previous resting-state fMRI studies have focused on and revealed brain functional changes in the default mode network (DMN). Interruptions of DMN-connections and alterations of DMN-seeded whole-brain connections, including both reduced number of connections and affected connectivity strengths, have been reported in football players (12, 15), veterans (16), multiple contact-sports athletes (13), and mild TBI patients (21, 23, 24). Partially consistent with these resting-state fMRI reports, we also observe 16 significantly reduced sFC connections (out of 48 in the NBS cluster) in fighters that involve key regions of the DMN such as bilateral precuneus (51). However, among these 16 connections, mostly are DMN-region-to-whole-brain connections (*N* = 15), which indicate that in fighters with repetitive head trauma, sFC disruptions are not limited to the DMN, and future fMRI analysis in subjects with repetitive head trauma might be beneficial using a whole-brain analysis.

At the same time, our unbalanced sample-sizes between NC and all fighters (35 vs. 133), and the statistical differences of YOE (differed by 1.22 years) and races (lack of diverse representations in NC) between NC and fighters might limit our interpretations of the above results. Even though we have included YOE and race variables as covariates in all our analysis, potential premorbid effects cannot be completely ruled out by linear regression and might therefore bias our results of NC vs. Fighters. Future analysis with a larger sample of NC that fully matches fighters' demographics, especially with a more diverse race representation, are necessary to validate our findings.

### Static Functional Brain Changes Related to Impaired PSS Task Performances Within Fighters

Our analysis first demonstrates sFC changes in a subset of fighters with impaired performances during visual-perception and fine-motor tasks through the group comparison between non-impaired and impaired fighters. Our definition for the impaired fighters' group is consistent with previous studies (19, 20), where only fighters with standardized PSS and (or) PSY scores below 97.8% of the age and education matched general populations are classified as impaired fighters. In impaired fighters, our results show significant decreases in frontal-temporal, limbic-frontal, temporal-temporal, and temporal-limbic sFC connections, as compared to non-impaired fighters (Table 3). These affected lobe-lobe connections are always retained in significant NBS clusters irrespective of the initial p-thresholds. These paths connect regions that are known to be involved in processing speed, working memory, attention, and other executive functions including hippocampus, amygdala, middle temporal regions, and frontal areas (52). We also have observed significantly affected sFC in impaired fighters that connect parietal, occipital, and cingulate regions (Figure 3), which have been previously reported to be important for visual-spatial perception and visual-attention functions (52). These observed sFC abnormalities in impaired fighters are also in line with previously reported structural observations. With the same group definition of impaired and non-impaired fighters, diffusion-weighted MRI data have revealed widespread structural connectivity damages in regions of the hippocampus, frontal, and parietal cortices, cingulum, striatum, and occipital regions in impaired fighters' group (19, 20). Furthermore, previous studies in TBI patients have shown that injuries to axons during one severe head trauma might disrupt the exquisite timing of neuronal communication within and between brain regions and might therefore account for the post-traumatic cognitive and executive dysfunctions (5, 53, 54). In our impaired fighters, previously reported structural (20) and currently observed functional deficits among relevant cognitive and executive connections indicate that repetitive sub-concussive brain injuries might also produce axonal damages that ultimately affect the substrate by which brain regions communicate with each other and cause related attention, cognitive and executive dysfunctions.

One potential bias in the above group comparison results is that an artificial group-division of impairment within fighters are being created. From this perspective, the association analysis between sFC and PSS scores in all 133 fighters without group-division could supplement and further reveal sFC changes related to worse PSS task performances in all fighters. From this perspective, our association analysis finds 13 frontal and temporal oriented sFC connections that are common between the group-comparison result and association regression result, which demonstrate that fighters with worse performances on fine-motor tasks do show sFC deficits among frontal, temporal and limbic connections (Figure 4). In addition, the association analysis also reveals significant sFC deficits related to worse PSS scores in frontal-occipital and parietal-occipital connections, which are central to the visual-perception functions (52). These additional connections uncovered by the association analysis are also related to the observed significant differences between NC and fighters and might be partially explained by the different sensitivity of sFC changes to continuous PSS measures within all fighters in the regression analysis, as compared to the sensitivity of sFC changes to binary PSS-based group divisions in between-group comparisons.

### Non-Significant Dynamic Functional Changes

Our study is the first attempt to investigate how repetitive head trauma affects dynamic functional brain changes in fighters, and we choose the computationally efficient dFC indices that can assess the temporal variabilities of the whole-brain functional connections, i.e., the temporal variation across all windows in the sliding-window approach for each edge (28). However, we have not found any significant clusters showing dFC differences between NCs and fighters or between non-impaired and impaired fighters, after correction for multiple comparisons using NBS. Three reasons might contribute to the non-significant results: (1) the dFC measure adds a temporal variability layer to the sFC measure, and therefore has increased the chances of variations, as compared to the sFC measure; (2) our fighters' population is a mix of both impaired fighters and non-impaired fighters, and therefore, variations of the dFC measure might be too large to detect any significant between-group differences, especially after correction for 3,160 comparisons; and (3) the selected dFC measure of temporal variations across all windows might not be sensitive enough to detect subtle dFC changes in fighters with repetitive head trauma, as previous study using the same dFC measure has also failed to detect any significant differences between NC and TBI patients (14). However, at a trend-level of p_NBS−corr_ = 0.08, our analysis does find stronger temporal variabilities in impaired fighters than non-impaired fighters among regions such as cingulate cortex, medial temporal lobe, and striatum. These affected dFC still minimally demonstrate a medium effect size of |*d*| = 0.46. Both weaker sFC and structural damages have been observed among these regions from the current and previous reports (18, 55). Therefore, overall, from our analysis, it can only be concluded that dFC measure of temporal variations across all windows in the sliding-window approach might not be sensitive enough to uncover significant between-group differences in fighters with repetitive head trauma. Future analysis with various dFC measures such as dFC-states (32), and dFC-stabilities (31) might uncover a more comprehensive picture of dynamic functional brain changes in this fighters' population.

### Limitations and Future Studies

Several limitations should be considered for the current study. Foremost, as we have stated above, the significant YOE and race differences between NC and fighters might bias our results, and future analysis with a larger sample and of NCs that more comprehensively match all fighters' demographics are necessary to validate our findings. Additionally, in our statistical analysis, other potential contributing factors to sFC and dFC such as time to last severe brain injury, substance usage and mood status have not been explicitly controlled and future comprehensive analysis with their effects on functional brain changes is needed. Furthermore, no consensus has yet been reached on the best dynamic FC analysis methods in fMRI data analysis. Our choice of dFC variation across all windows is computationally efficient for our 168 subjects but might lack the sensitivity to detect subtle dynamic brain changes in fighters experiencing repetitive head hits. Since dynamic FC measures have been demonstrated in many other neurological conditions to be more sensitive to deficits than static FC measures (29), and our sFC results demonstrate evident deficits related to worse performance in PSS tasks with visual-attention and working-memory demands in fighters, future studies with more sensitive dFC measures (might also be more computationally involved) are warranted to further investigate functional brain changes in these fighters.

## Conclusion

In conclusion, using resting-state fMRI data, our study identifies weaker frontal-temporal, frontal-occipital, temporal-occipital, and parietal-occipital sFC connections in active professional fighters than in NC, which are in line with previous structural findings in subjects experiencing repetitive head hits (contact sports athletes, veterans, and mild TBI patients). In a subset of fighters with impaired-range performances on processing speed tasks with visual-perception, visual-attention and working-memory demands, significantly decreased sFC strengths are found among key regions involved in visual-attention, executive and cognitive process, as compared to non-impaired fighters. Without creating the artificial impairment group division in fighters, our association analysis also reveals similar sFC deficits to worse PSS task performances in all 133 fighters. With our choice of dFC indices, our study does not find any significant dynamic functional brain changes in fighters beyond a trend-level increased temporal variability among similar regions with weaker sFC strengths in impaired fighters.

## Data Availability Statement

The raw data supporting the conclusions of this article will be made available upon reasonable requests through PFBHS.

## Ethics Statement

The studies involving human participants were reviewed and approved by Cleveland Clinic Institutional Review Board. The patients/participants provided their written informed consent to participate in this study.

## Author Contributions

XZ and DC: conception and design of the study. XZ, VM, RN, ZY, KS, LB, and DC: analysis and interpretation of data. XZ, VM, CB, and DC: data management and quality control. XZ: drafting the article. XZ, VM, RN, ZY, KS, LB, CB, and DC: revising it critically for important intellectual content and final approval of the version to be submitted. All authors contributed to the article and approved the submitted version.

## Conflict of Interest

The authors declare that the research was conducted in the absence of any commercial or financial relationships that could be construed as a potential conflict of interest.
