# Supplementary material for: Resting-State Static and Dynamic Functional Abnormalities in Active Professional Fighters With Repetitive Head Trauma and With Neuropsychological Impairments
Source: Front Neurol. 2020 Dec 10;11:602586. doi: 10.3389/fneur.2020.602586 (PMC7758536; doi:10.3389/fneur.2020.602586)
Supplement: Supplementary file 1 [file Data_Sheet_1.docx]

**Supplementary for: Resting-state static and dynamic functional abnormalities in active professional fighters with repetitive head trauma and with neuropsychological impairments**

Xiaowei Zhuang^1,2^, Virendra Mishra^1^, Rajesh Nandy^3^, Zhengshi Yang^1,2^, Karthik Sreenivasan^1,2^, Lauren Bennett^4^, Charles Bernick^1, 5^, Dietmar Cordes^1, 2, 6^

^1^ Cleveland Clinic Lou Ruvo Center for Brain Health, Las Vegas, Nevada, USA

^2^ Department of Brain Health, University of Nevada, Las Vegas, Nevada, USA.

^3^ University of North Texas, Fort Worth, TX, USA

^4^ Pickup Family Neuroscience Institute, Hoag Memorial Hospital Presbyterian, Newport Beach, California, USA

^5^ UW Medicine, Seattle, Washington, USA

^6^ University of Colorado, Boulder, Colorado, USA

**Supplementary Tables:**

| Left Hemisphere | | | Right Hemisphere | | |
| --- | --- | --- | --- | --- | --- |
| Regions | Abbreviations | Lobe | Regions | Abbreviations | Lobe |
| lh-caudalmiddlefrontal | L MF-caud | Frontal | rh-caudalmiddlefrontal | R MF-caud | Frontal |
| lh-lateralorbitofrontal | L LOFC | Frontal | rh-lateralorbitofrontal | R LOFC | Frontal |
| lh-medialorbitofrontal | L MOFC | Frontal | rh-medialorbitofrontal | R MOFC | Frontal |
| lh-paracentral | L Paracen | Frontal | rh-paracentral | R Paracen | Frontal |
| lh-parsopercularis | L Pars-oper | Frontal | rh-parsopercularis | R Pars-oper | Frontal |
| lh-parsorbitalis | L Pars-orbi | Frontal | rh-parsorbitalis | R Pars-orbi | Frontal |
| lh-parstriangularis | L Pars-tria | Frontal | rh-parstriangularis | R Pars-tria | Frontal |
| lh-precentral | L Precen | Frontal | rh-precentral | R Precen | Frontal |
| lh-rostralmiddlefrontal | L MFG-rost | Frontal | rh-rostralmiddlefrontal | R MFG-rost | Frontal |
| lh-superiorfrontal | L SFG | Frontal | rh-superiorfrontal | R SFG | Frontal |
| lh-frontalpole | L FP | Frontal | rh-frontalpole | R FP | Frontal |
| lh-insula | L Insula | Insula | rh-insula | R Insula | Insula |
| lh-Hippocampus | L HIPP | Limbic | rh-Hippocampus | R HIPP | Limbic |
| lh-Amygdala | L AMYG | Limbic | rh-Amygdala | R AMYG | Limbic |
| lh-cuneus | L Ceneus | Occipital | rh-cuneus | R Ceneus | Occipital |
| lh-lateraloccipital | L LOCC | Occipital | rh-lateraloccipital | R LOCC | Occipital |
| lh-lingual | L Ling | Occipital | rh-lingual | R Ling | Occipital |
| lh-pericalcarine | L PeriCal | Occipital | rh-pericalcarine | R PeriCal | Occipital |
| lh-inferiorparietal | L IPC | Parietal | rh-inferiorparietal | R IPC | Parietal |
| lh-postcentral | L Postcen | Parietal | rh-postcentral | R Postcen | Parietal |
| lh-precuneus | L Precuneus | Parietal | rh-precuneus | R Precuneus | Parietal |
| lh-superiorparietal | L SPC | Parietal | rh-superiorparietal | R SPC | Parietal |
| lh-supramarginal | L SMG | Parietal | rh-supramarginal | R SMG | Parietal |
| lh-Thalamus | L THAL | sub-cortical | rh-Thalamus | R THAM | sub-cortical |
| lh-Caudate | L CAUD | sub-cortical | rh-Caudate | R CAUD | sub-cortical |
| lh-Putamen | L PUTA | sub-cortical | rh-Putamen | R PUTA | sub-cortical |
| lh-Pallidum | L PALL | sub-cortical | rh-Pallidum | R PALL | sub-cortical |
| lh-bankssts | L Bankssts | Temporal | rh-bankssts | R Bankssts | Temporal |
| lh-entorhinal | L ERC | Temporal | rh-entorhinal | R ERC | Temporal |
| lh-fusiform | L FUS | Temporal | rh-fusiform | R FUS | Temporal |
| lh-inferiortemporal | L ITG | Temporal | rh-inferiortemporal | R ITG | Temporal |
| lh-middletemporal | L MTG | Temporal | rh-middletemporal | R MTG | Temporal |
| lh-parahippocampal | L PHG | Temporal | rh-parahippocampal | R PHG | Temporal |
| lh-superiortemporal | L STG | Temporal | rh-superiortemporal | R STG | Temporal |
| lh-temporalpole | L TP | Temporal | rh-temporalpole | R TP | Temporal |
| lh-transversetemporal | L TTG | Temporal | rh-transversetemporal | R TTG | Temporal |
| lh-caudalanteriorcingulate | L ACC-caud | CingulateCortex | rh-caudalanteriorcingulate | R ACC-caud | CingulateCortex |
| lh-isthmuscingulate | L CC-isth | CingulateCortex | rh-isthmuscingulate | R CC-isth | CingulateCortex |
| lh-posteriorcingulate | L PCC | CingulateCortex | rh-posteriorcingulate | R PCC | CingulateCortex |
| lh-rostralanteriorcingulate | L ACC-rost | CingulateCortex | rh-rostralanteriorcingulate | R ACC-rost | CingulateCortex |

Table S1. Regions of interest (ROIs) from Desikan-Killiany atlas and the subcortical labeling. The abbreviations lh and rh represent left and right hemispheres, respectively.

|  | Nonimpaired Fighters | Impaired Fighters | Group difference |
| --- | --- | --- | --- |
| Reaction Time (Total) | 684.49±71.82 | 761.25±115.10 | **<0.001** |
| Verbal Memory (Total) | 51.35±5.05 | 50.66±5.31 | 0.45 |
| Verbal Memory (Correct Hits Immediate Recall) | 11.78±2.38 | 11.55±2.80 | 0.63 |
| Verbal Memory (Correct Passes Immediate Recall) | 14.35±1.00 | 14.38±1.03 | 0.84 |
| Verbal Memory (Correct Hits Delayed Recall) | 11.13±2.39 | 10.54±2.88 | 0.21 |
| Verbal Memory (Correct Passes Delayed Recall) | 14.10±1.38 | 14.18±1.16 | 0.69 |
| Stroop (Commission Errors) | 1.28±1.53 | 1.84±1.86 | 0.06 |
| Stroop (Simple Reaction Time) | 299.82±76.60 | 340.69±87.51 | **0.01** |
| Stroop (Complex Reaction Time) | 637.00±69.02 | 699.54±135.00 | **0.001** |
| Stroop (Reaction Time Total) | 730.11±98.01 | 822.36±133.62 | **<0.001** |

Table S2. 10 scores output from the verbal memory and Stroop tasks from the CNS Vital Signs in each fighters’ groups. These 10 scores were used as features to classify and validate the fighters’ impairment status, which were determined from the PSS and PSY scores. A classification accuracy of 63.91% is obtained, which exceed the 95^th^ percentile of the permutation accuracy (59.65%).


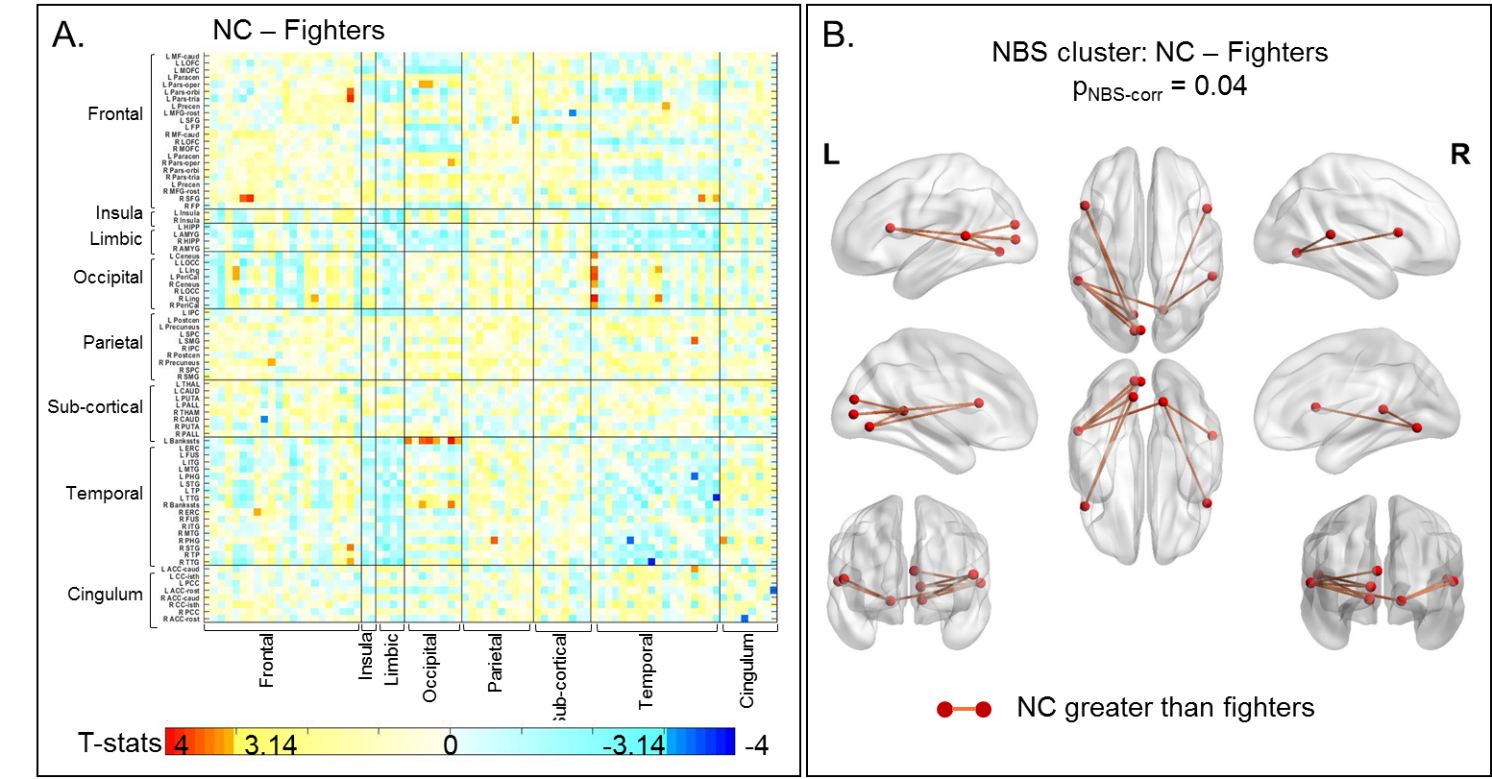


Fig. S1. sFC comparisons between NC and fighters using uncorrected p-value of 0.001 in NBS.

***(A)*** Uncorrected t-statistic map for contrast $\boldsymbol{c}_{1}:$NC-fighters. Uncorrected p-value p_unc_<0.001 is used in NBS step1 to form supra-threshold clusters for contrast NC greater than fighters, and fighters greater than NC, respectively. ***(B)*** 8 Stronger connections within the significant NBS cluster in NCs than in fighters, after correction for multiple comparisons (p_NBS-corr_=0.04). These are mainly frontal-occipital (37.5%) and temporal-occipital (62.5%) connections. The red circles and sticks represent the nodes and edges connecting the nodes, respectively. The nodes and edges are visualized on the Desikan-Killiany template. Images are shown in neurological convention.

| sFC connections | | Cohen's $d$ | Uncorrected p-values |
| --- | --- | --- | --- |
| ROI 1 | ROI 2 |  |  |
| **lh-bankssts** | **rh-lingual** | **0.78** | **6.32E-05** |
| rh-superiorfrontal | lh-parstriangularis | 0.76 | 9.55E-05 |
| **lh-bankssts** | **lh-pericalcarine** | **0.72** | **1.98E-04** |
| **lh-bankssts** | **lh-lingual** | **0.70** | **2.66E-04** |
| rh-parahippocampal | lh-supramarginal | 0.70 | 2.68E-04 |
| rh-superiorfrontal | lh-parsorbitalis | 0.70 | 2.76E-04 |
| **lh-bankssts** | **lh-cuneus** | **0.68** | **4.21E-04** |
| rh-superiortemporal | rh-superiorfrontal | 0.66 | 5.25E-04 |
| **rh-bankssts** | **rh-lingual** | **0.66** | **5.56E-04** |
| lh-caudalanteriorcingulate | rh-parahippocampal | 0.65 | 6.93E-04 |
| **lh-pericalcarine** | **lh-parsopercularis** | **0.63** | **9.09E-04** |
| **rh-lingual** | **rh-parsopercularis** | **0.63** | **9.56E-04** |
| **lh-lingual** | **lh-parsopercularis** | **0.63** | **9.87E-04** |
| rh-transversetemporal | rh-superiorfrontal | 0.62 | 1.09E-03 |
| lh-bankssts | rh-cuneus | 0.62 | 1.17E-03 |
| rh-precuneus | lh-superiorfrontal | 0.61 | 1.22E-03 |
| lh-bankssts | rh-pericalcarine | 0.61 | 1.28E-03 |
| rh-bankssts | lh-lingual | 0.61 | 1.35E-03 |
| lh-transversetemporal | rh-superiorfrontal | 0.60 | 1.49E-03 |
| lh-superiortemporal | rh-superiorfrontal | 0.60 | 1.58E-03 |
| rh-lingual | lh-parsopercularis | 0.60 | 1.61E-03 |
| lh-bankssts | lh-paracentral | 0.59 | 1.81E-03 |
| lh-bankssts | rh-paracentral | 0.59 | 1.89E-03 |
| lh-Putamen | rh-caudalmiddlefrontal | 0.58 | 1.97E-03 |
| lh-inferiortemporal | lh-precuneus | 0.58 | 2.01E-03 |
| rh-supramarginal | rh-lingual | 0.58 | 2.12E-03 |
| lh-inferiortemporal | rh-precuneus | 0.57 | 2.25E-03 |
| lh-bankssts | rh-superiorfrontal | 0.57 | 2.51E-03 |
| rh-postcentral | rh-cuneus | 0.57 | 2.54E-03 |
| lh-pericalcarine | rh-parstriangularis | 0.56 | 2.72E-03 |
| rh-lingual | rh-parstriangularis | 0.56 | 2.83E-03 |
| lh-supramarginal | rh-lingual | 0.56 | 2.91E-03 |
| lh-insula | rh-superiorfrontal | 0.56 | 2.93E-03 |
| rh-supramarginal | rh-pericalcarine | 0.56 | 2.97E-03 |
| rh-postcentral | rh-lingual | 0.55 | 3.00E-03 |
| rh-superiortemporal | lh-superiorfrontal | 0.55 | 3.37E-03 |
| rh-bankssts | rh-superiorfrontal | 0.55 | 3.45E-03 |
| lh-transversetemporal | rh-lingual | 0.54 | 3.77E-03 |
| rh-Pallidum | rh-caudalmiddlefrontal | 0.54 | 3.89E-03 |
| rh-pericalcarine | rh-parsopercularis | 0.54 | 3.91E-03 |
| lh-insula | rh-caudalmiddlefrontal | 0.54 | 3.93E-03 |
| rh-superiortemporal | rh-precentral | 0.54 | 3.97E-03 |
| lh-lingual | rh-parstriangularis | 0.54 | 3.98E-03 |
| rh-superiortemporal | rh-paracentral | 0.53 | 4.13E-03 |
| lh-caudalanteriorcingulate | lh-parahippocampal | 0.53 | 4.44E-03 |
| rh-isthmuscingulate | lh-inferiortemporal | 0.53 | 4.49E-03 |
| rh-caudalmiddlefrontal | lh-parstriangularis | 0.53 | 4.56E-03 |
| rh-lingual | rh-precentral | 0.53 | 4.60E-03 |

Table S3. Static functional connections (sFC) in the cluster that are significantly stronger in NCs than in fighters after NBS correction for multiple comparisons. Connections in the significant NBS cluster using initial uncorrected p-value of 0.005 are shown, and connections in the significant NBS cluster using initial uncorrected p-values of 0.001 are highlighted in red and bold. Corresponding effect sizes (column 3) and uncorrected p-values (column 4) for each connection are also listed. The abbreviations lh and rh represent left and right hemispheres, respectively.

**
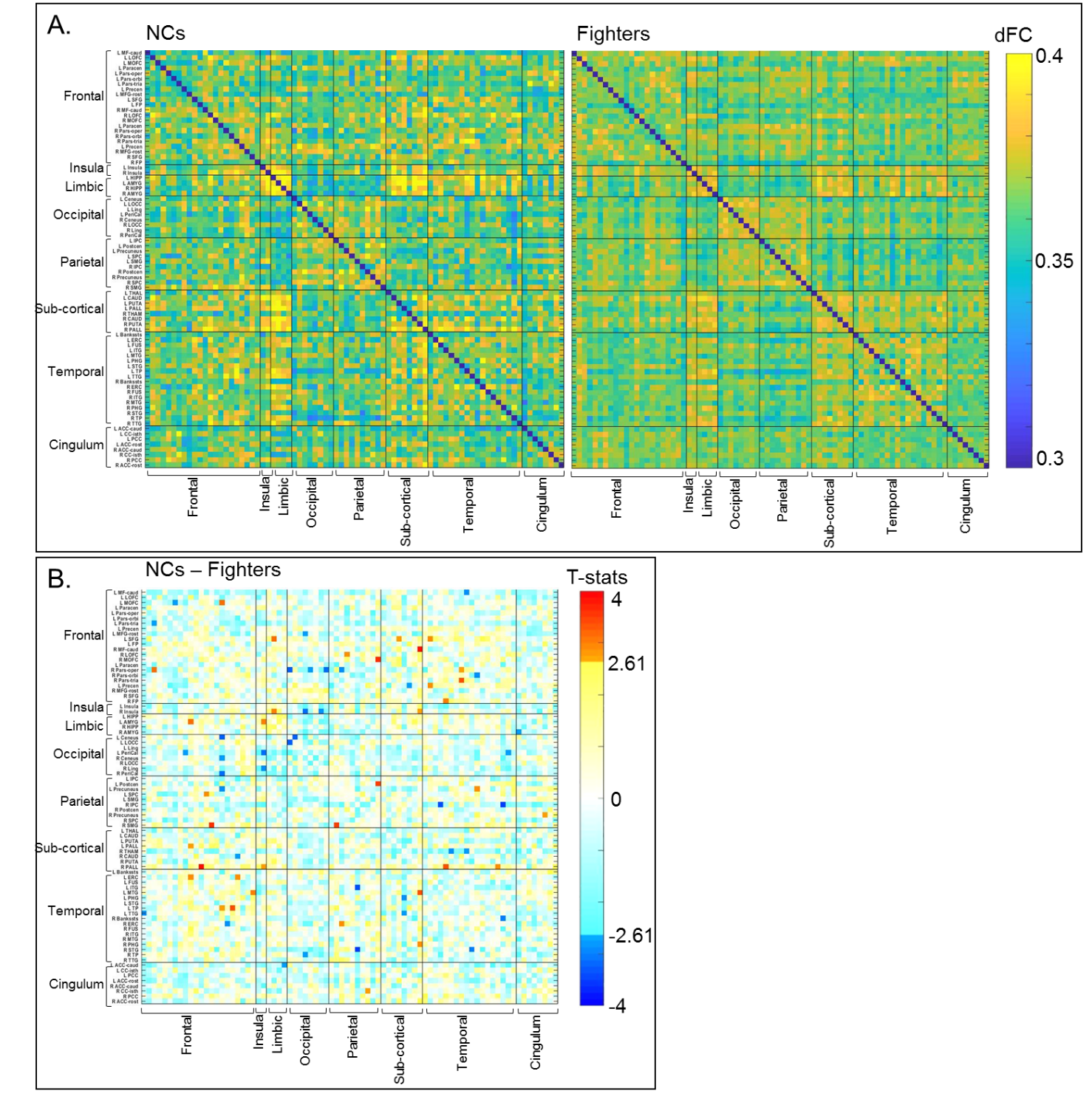
**

## Fig. S2. dFC comparison between NCs and all fighters.

(A) Average dFC matrices (pair-wise variabilities) for NCs and fighters. (B) Uncorrected t-statistic map for contrast$\boldsymbol{c}_{1}$: NC vs. fighters.


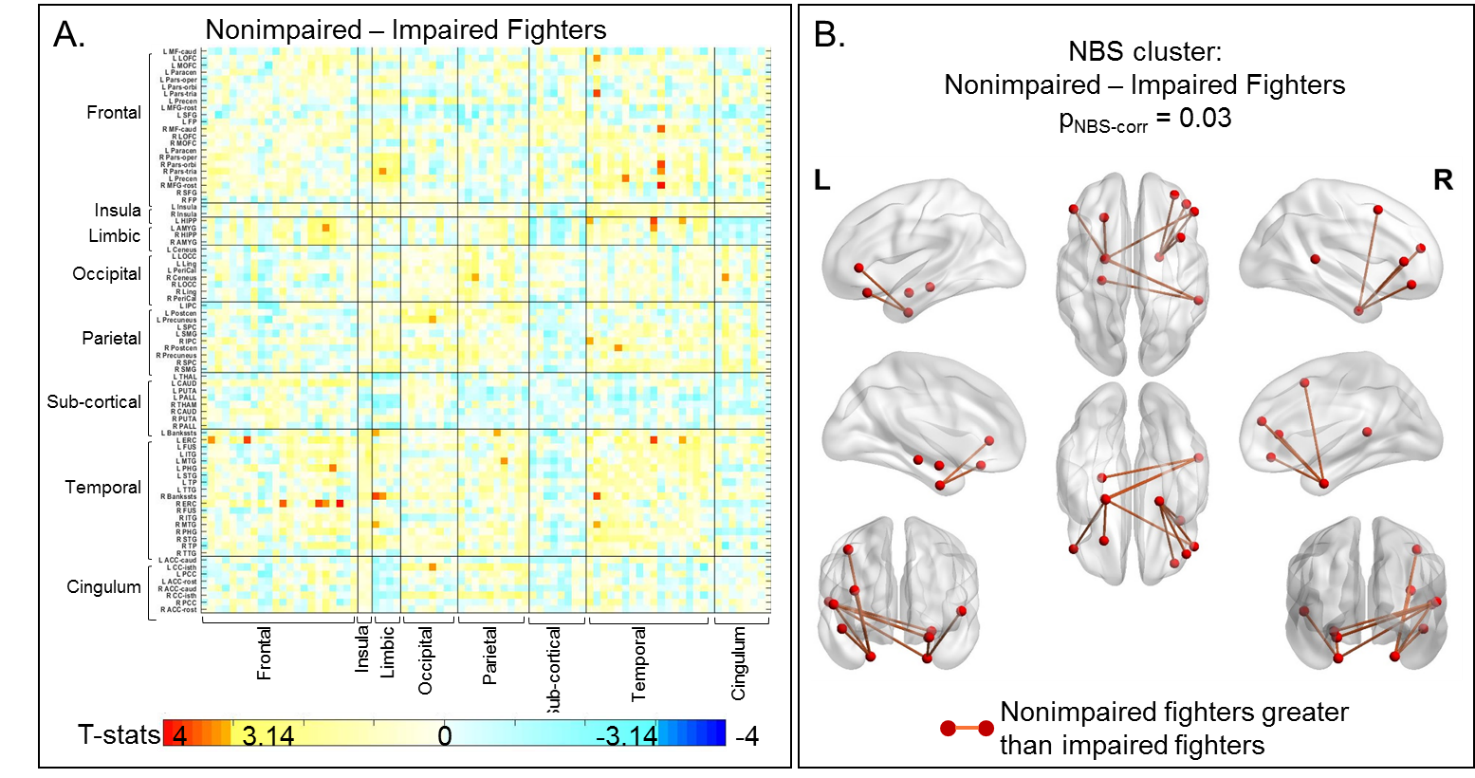


Fig. S3. sFC comparisons between nonimpaired and impaired fighters using uncorrected p-value of 0.001 in NBS.

***(A)*** Uncorrected t-statistic map for contrast $\boldsymbol{c}_{2}:$nonimpaired fighters-impaired fighters. Uncorrected p-value p_unc_<0.001 is used in NBS step1 to form supra-threshold clusters for contrast nonimpaired greater than impaired fighters, and impaired fighters greater than nonimpaired fighters in NBS, respectively. ***(B)*** 10 Stronger connections within the significant NBS cluster (p_NBS-corr_=0.03) in nonimpaired fighters than in impaired fighters. These are mainly temporal-frontal (60%), temporal-limbic (20%), limbic-frontal (10%), and temporal-temporal (10%) connections. The red circles and sticks represent the nodes and edges connecting the nodes, respectively. The nodes and edges are visualized on the Desikan-Killiany template. Images are shown in neurological convention.

| sFC connections | | Cohen's $d$ | Uncorrected p-values |
| --- | --- | --- | --- |
| ROI 1 | ROI 2 |  |  |
| **rh-entorhinal** | **rh-rostralmiddlefrontal** | **0.80** | **5.46E-06** |
| **lh-entorhinal** | **lh-parstriangularis** | **0.65** | **1.37E-04** |
| **rh-bankssts** | **lh-Hippocampus** | **0.65** | **1.61E-04** |
| **rh-bankssts** | **lh-entorhinal** | **0.64** | **1.79E-04** |
| **rh-entorhinal** | **rh-parsorbitalis** | **0.63** | **2.00E-04** |
| **rh-entorhinal** | **rh-caudalmiddlefrontal** | **0.62** | **2.72E-04** |
| lh-parahippocampal | rh-precentral | 0.60 | 3.95E-04 |
| **lh-entorhinal** | **lh-lateralorbitofrontal** | **0.58** | **5.79E-04** |
| lh-middletemporal | rh-postcentral | 0.58 | 6.21E-04 |
| **rh-bankssts** | **lh-Amygdala** | **0.58** | **6.30E-04** |
| **rh-entorhinal** | **rh-parstriangularis** | **0.57** | **6.85E-04** |
| **lh-Amygdala** | **rh-parstriangularis** | **0.57** | **6.94E-04** |
| lh-isthmuscingulate | rh-cuneus | 0.57 | 7.68E-04 |
| lh-bankssts | lh-Hippocampus | 0.55 | 1.06E-03 |
| rh-middletemporal | lh-entorhinal | 0.54 | 1.26E-03 |
| lh-bankssts | rh-inferiorparietal | 0.54 | 1.26E-03 |
| lh-precuneus | rh-cuneus | 0.53 | 1.37E-03 |
| rh-middletemporal | lh-Hippocampus | 0.53 | 1.40E-03 |
| rh-Hippocampus | rh-precentral | 0.53 | 1.56E-03 |
| rh-isthmuscingulate | rh-cuneus | 0.52 | 1.68E-03 |
| lh-postcentral | rh-cuneus | 0.52 | 1.79E-03 |
| rh-superiortemporal | lh-entorhinal | 0.52 | 1.84E-03 |
| lh-entorhinal | rh-rostralmiddlefrontal | 0.52 | 1.87E-03 |
| rh-parahippocampal | rh-precentral | 0.51 | 1.95E-03 |
| rh-isthmuscingulate | lh-supramarginal | 0.51 | 1.97E-03 |
| lh-entorhinal | lh-bankssts | 0.51 | 1.99E-03 |
| lh-entorhinal | rh-parsorbitalis | 0.51 | 2.00E-03 |
| lh-posteriorcingulate | rh-parstriangularis | 0.51 | 2.10E-03 |
| rh-postcentral | rh-cuneus | 0.51 | 2.22E-03 |
| lh-middletemporal | lh-entorhinal | 0.50 | 2.53E-03 |
| lh-Hippocampus | rh-parstriangularis | 0.50 | 2.60E-03 |
| lh-isthmuscingulate | lh-supramarginal | 0.48 | 3.26E-03 |
| lh-postcentral | lh-cuneus | 0.48 | 3.30E-03 |
| lh-Amygdala | rh-parsopercularis | 0.48 | 3.32E-03 |
| rh-isthmuscingulate | rh-superiorparietal | 0.48 | 3.34E-03 |
| lh-Caudate | rh-precentral | 0.48 | 3.41E-03 |
| rh-precuneus | lh-precuneus | 0.48 | 3.64E-03 |
| lh-entorhinal | rh-parstriangularis | 0.47 | 3.85E-03 |
| lh-entorhinal | lh-parsopercularis | 0.47 | 3.91E-03 |
| lh-Hippocampus | rh-precentral | 0.47 | 3.94E-03 |
| rh-entorhinal | lh-rostralmiddlefrontal | 0.47 | 3.95E-03 |
| rh-Amygdala | rh-parstriangularis | 0.46 | 4.55E-03 |
| lh-precuneus | lh-lateraloccipital | 0.46 | 4.65E-03 |
| lh-precuneus | lh-pericalcarine | 0.46 | 4.77E-03 |
| lh-parahippocampal | lh-bankssts | 0.46 | 4.81E-03 |

Table S4. sFC connections in the cluster that are significantly stronger in nonimpaired fighters than in impaired fighters after NBS correction for multiple comparisons. Connections in the significant NBS cluster using initial uncorrected p-value of 0.005 are shown, and connections in the significant NBS cluster using initial uncorrected p-value of 0.001 are highlighted in red and bold. Corresponding effect sizes (column 3) and uncorrected p-values (column 4) for each connection are also listed. The abbreviations lh and rh represent left and right hemispheres, respectively.


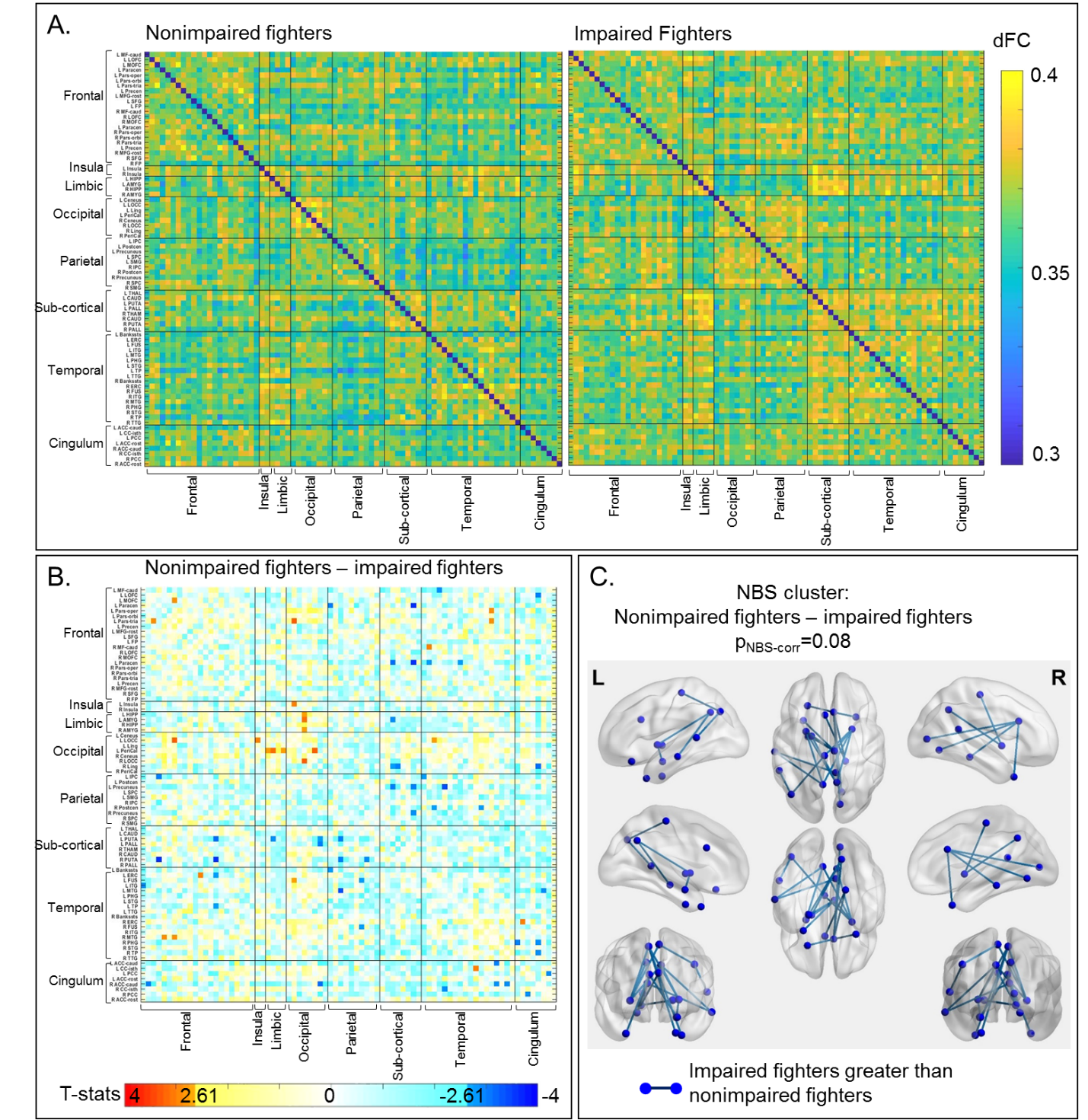


## Fig. S4. dFC comparison between nonimpaired and impaired fighters using uncorrected p-value of 0.005 in NBS.

***(A)*** Average dFC matrices (pair-wise variabilities) for nonmpaired and impaired fighters. ***(B)*** Uncorrected t-statistic map for contrast $\boldsymbol{c}_{2}:$nonimpaired fighters - impaired fighters. T=2.61 (uncorrected p=0.005, one tail threshold for t-test with the degree of freedom of 160) is used in NBS step1 to form supra-threshold clusters for NC greater than fighters, and T=-2.61 is used to form supra-threshold clusters for the reversed contrast in NBS. ***(C)*** Weaker connections within the trend level significant NBS cluster (p_NBS-corr_=0.08) in nonimpaired fighters than in impaired fighters, i.e. for reversed contrast. These are mainly temporal-cingulate cortex, sub-cortical-frontal, and sub-cortical-parietal connections. The blue circles and sticks represent the nodes and edges connecting the nodes, respectively. The nodes and edges are visualized on the Desikan-Killiany template, the same as both axis in *(A)* and *(B)*. Images are shown in neurological convention.

| dFC connections | | Cohen's $d$ | Uncorrected p-values |
| --- | --- | --- | --- |
| ROI 1 | ROI 2 |  |  |
| rh-Putamen | rh-paracentral | -0.63 | 2.19E-04 |
| rh-Putamen | lh-paracentral | -0.63 | 2.35E-04 |
| lh-middletemporal | lh-precuneus | -0.59 | 5.33E-04 |
| rh-caudalanteriorcingulate | rh-lingual | -0.55 | 1.11E-03 |
| lh-Putamen | rh-paracentral | -0.54 | 1.24E-03 |
| rh-caudalanteriorcingulate | lh-lateralorbitofrontal | -0.53 | 1.61E-03 |
| lh-Putamen | lh-precuneus | -0.52 | 1.64E-03 |
| lh-inferiorparietal | lh-paracentral | -0.52 | 1.79E-03 |
| rh-Putamen | lh-precuneus | -0.52 | 1.88E-03 |
| lh-caudalanteriorcingulate | rh-parahippocampal | -0.52 | 1.92E-03 |
| lh-Pallidum | rh-lingual | -0.51 | 1.94E-03 |
| lh-Putamen | lh-Amygdala | -0.50 | 2.36E-03 |
| rh-caudalanteriorcingulate | rh-temporalpole | -0.49 | 3.06E-03 |
| rh-isthmuscingulate | lh-middletemporal | -0.49 | 3.16E-03 |
| rh-caudalanteriorcingulate | rh-parahippocampal | -0.48 | 3.27E-03 |
| rh-Putamen | rh-precuneus | -0.48 | 3.28E-03 |
| lh-temporalpole | rh-paracentral | -0.47 | 3.86E-03 |
| lh-entorhinal | rh-paracentral | -0.47 | 4.36E-03 |
| lh-bankssts | lh-inferiorparietal | -0.47 | 4.38E-03 |
| lh-Putamen | rh-lingual | -0.46 | 4.66E-03 |
| rh-caudalanteriorcingulate | rh-pericalcarine | -0.46 | 4.84E-03 |

Table S5. Dynamic functional connections (dFC) in the cluster that are at trend level and more varying temporally in impaired fighters than in nonimpaired fighters after NBS correction for multiple comparisons (p_NBS-corr_=0.08). Corresponding effect sizes (column 3) and uncorrected p-values (column 4) for each connection are also listed. The abbreviations lh and rh represent left and right hemispheres, respectively.

**
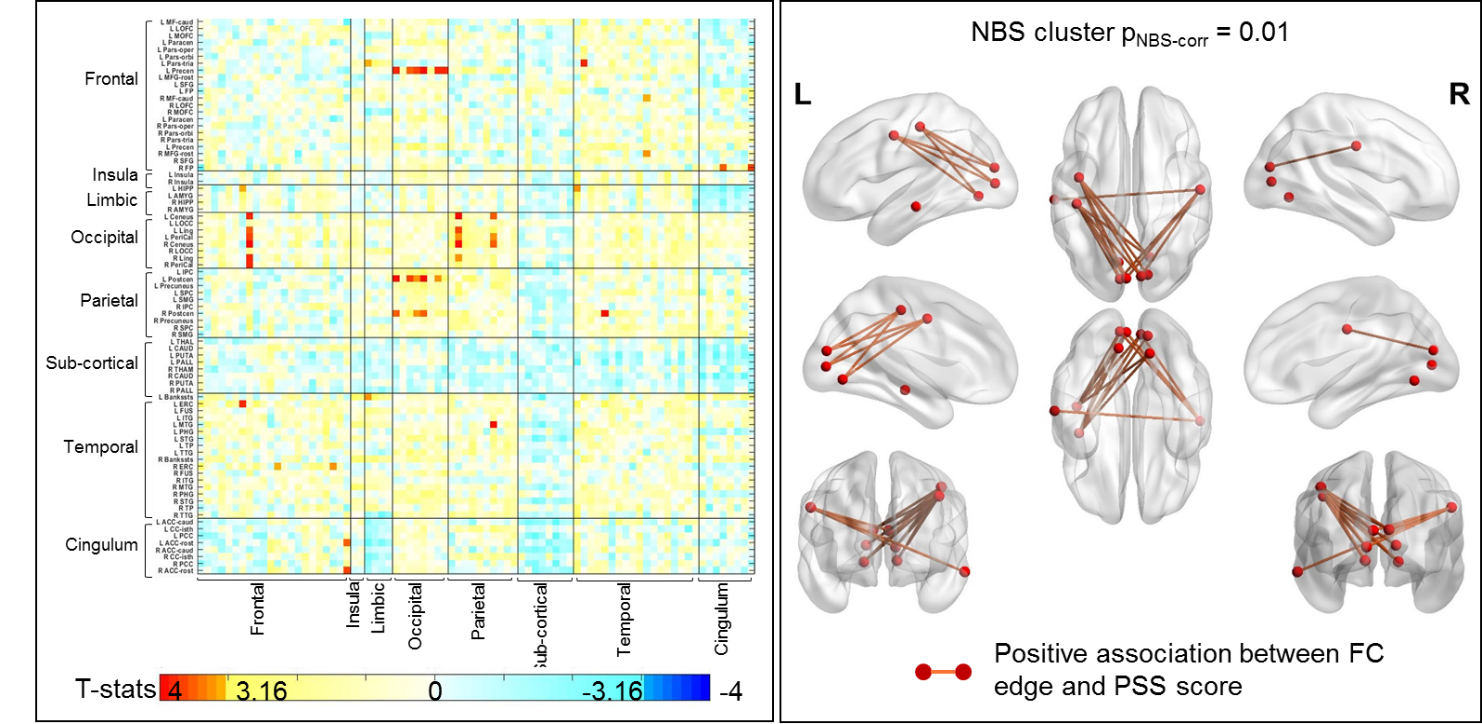
**

Fig. S5. sFC association with PSS score in all 133 fighters with uncorrected p-value of 0.001 in the NBS.

*(A).* Uncorrected t-statistic map for association between sFC and PSS score. Uncorrected p-value<0.001 is used in NBS step1 to form supra-threshold clusters for the positive and negative associations separately. *(B).* 15 sFC connections within the significant NBS cluster (p_NBS-corr_=0.01) that shows positive association between sFC and PSS scores. These are mainly parietal-occipital and occipital-frontal connections, with regions involved in visual-perception and executive functions. The red circles and sticks represent the nodes and edges connecting the nodes, respectively. The nodes and edges are visualized on the Desikan-Killiany template. Images are shown in neurological convention.

| sFC connections | | Uncorrected p-value | Common with group-comparison |
| --- | --- | --- | --- |
| ROI 1 | ROI 2 |  |  |
| **rh-cuneus** | **lh-postcentral** | **1.59E-05** | Common |
| **lh-cuneus** | **lh-postcentral** | **2.21E-05** | Common |
| **lh-precentral** | **rh-cuneus** | **4.12E-05** |  |
| **rh-postcentral** | **lh-middletemporal** | **6.75E-05** | Common |
| **lh-precentral** | **lh-cuneus** | **9.50E-05** |  |
| **lh-precentral** | **rh-lingual** | **1.07E-04** |  |
| **lh-precentral** | **rh-pericalcarine** | **1.13E-04** |  |
| lh-parstriangularis | lh-entorhinal | 1.15E-04 | Common |
| **lh-precentral** | **lh-pericalcarine** | **1.50E-04** |  |
| **lh-lingual** | **lh-postcentral** | **1.96E-04** |  |
| **lh-precentral** | **lh-lingual** | **2.54E-04** |  |
| **rh-cuneus** | **rh-postcentral** | **3.57E-04** | Common |
| **lh-cuneus** | **rh-postcentral** | **3.83E-04** |  |
| **lh-pericalcarine** | **lh-postcentral** | **8.06E-04** |  |
| **lh-pericalcarine** | **rh-postcentral** | **8.12E-04** |  |
| **rh-lingual** | **lh-postcentral** | **8.19E-04** |  |
| lh-Hippocampus | lh-bankssts | 8.42E-04 | Common |
| rh-rostralmiddlefrontal | rh-entorhinal | 9.13E-04 | Common |
| lh-parstriangularis | lh-Hippocampus | 1.28E-03 |  |
| rh-caudalmiddlefrontal | rh-entorhinal | 1.30E-03 | Common |
| rh-cuneus | lh-inferiorparietal | 1.48E-03 |  |
| lh-caudalmiddlefrontal | rh-entorhinal | 1.60E-03 |  |
| rh-precentral | lh-pericalcarine | 1.70E-03 |  |
| lh-bankssts | lh-entorhinal | 1.70E-03 | Common |
| lh-entorhinal | lh-middletemporal | 1.76E-03 | Common |
| lh-caudalmiddlefrontal | lh-entorhinal | 1.80E-03 |  |
| lh-insula | lh-bankssts | 2.13E-03 |  |
| lh-lingual | rh-postcentral | 2.22E-03 |  |
| lh-insula | rh-middletemporal | 2.67E-03 |  |
| lh-postcentral | lh-superiorparietal | 2.67E-03 |  |
| lh-bankssts | rh-transversetemporal | 2.98E-03 |  |
| lh-Amygdala | lh-bankssts | 3.02E-03 |  |
| lh-parstriangularis | lh-Amygdala | 3.46E-03 |  |
| rh-pericalcarine | lh-postcentral | 3.54E-03 |  |
| rh-cuneus | lh-precuneus | 3.55E-03 | Common |
| lh-parsopercularis | lh-entorhinal | 3.59E-03 | Common |
| lh-bankssts | rh-temporalpole | 3.76E-03 |  |
| lh-lingual | lh-transversetemporal | 4.40E-03 |  |
| lh-rostralmiddlefrontal | lh-entorhinal | 4.53E-03 |  |
| rh-precentral | lh-parahippocampal | 4.80E-03 | Common |

Table S6. sFC connections in the cluster that are significantly associated with PSS scores in all fighters after NBS correction for multiple comparisons. Connections in the significant NBS cluster using initial uncorrected p-value of 0.005 are shown, and connections in the significant NBS cluster using initial uncorrected p-values of 0.001 are highlighted in red and bold. Common significant connections between association analysis and group-comparison between nonimpaired and impaired fighters are indicated in column 4. The abbreviations lh and rh represent left and right hemispheres, respectively.
